# Using nanoBRET and CRISPR/Cas9 to monitor proximity to a genome-edited protein in real-time

**DOI:** 10.1038/s41598-017-03486-2

**Published:** 2017-06-09

**Authors:** Carl W. White, Hannah K. Vanyai, Heng B. See, Elizabeth K. M. Johnstone, Kevin D. G. Pfleger

**Affiliations:** 1 Molecular Endocrinology and Pharmacology, Harry Perkins Institute of Medical Research, QEII Medical Centre, Nedlands, Western Australia 6009 Australia; 2 Centre for Medical Research, The University of Western Australia, Crawley, Western Australia 6009 Australia; 3 Brain Growth and Disease Laboratory, Harry Perkins Institute of Medical Research, QEII Medical Centre, Nedlands, Western Australia 6009 Australia; 4 Dimerix Limited, Nedlands, Western Australia 6009 Australia

## Abstract

Bioluminescence resonance energy transfer (BRET) has been a vital tool for understanding G protein-coupled receptor (GPCR) function. It has been used to investigate GPCR-protein and/or -ligand interactions as well as GPCR oligomerisation. However the utility of BRET is limited by the requirement that the fusion proteins, and in particular the donor, need to be exogenously expressed. To address this, we have used CRISPR/Cas9-mediated homology-directed repair to generate protein-Nanoluciferase (Nluc) fusions under endogenous promotion, thus allowing investigation of proximity between the genome-edited protein and an exogenously expressed protein by BRET. Here we report BRET monitoring of GPCR-mediated β-arrestin2 recruitment and internalisation where the donor luciferase was under endogenous promotion, in live cells and in real time. We have investigated the utility of CRISPR/Cas9 genome editing to create genome-edited fusion proteins that can be used as BRET donors and propose that this strategy can be used to overcome the need for exogenous donor expression.

## Introduction

G protein-coupled receptors (GPCRs) are a large family of membrane bound proteins responsible for signal transduction. By binding an array of structurally diverse natural ligands, GPCRs control and modulate essential physiological functions and have therefore been extensively targeted for the treatment of disease. Typically, agonist binding to a GPCR results in the stabilisation of a conformation that mediates the recruitment and activation of heterotrimeric G proteins required for the activation of intracellular signalling pathways. Following activation, phosphorylated GPCRs form further protein-protein interactions with numerous proteins including β-arrestin2, which results in desensitisation of G protein-mediated signalling at the plasma membrane and internalisation of the receptor via interaction with the clathrin machinery, as well as additional signalling via ‘non-classical’ pathways^1^. Additionally, β-arrestins appear to coordinate some of the spatiotemporal aspects of GPCR signalling^2, 3^. GPCR-protein interactions thus provide the key mechanism by which GPCRs function. Bioluminescence resonance energy transfer (BRET) is the non-radiative transfer of energy between a bioluminescent donor and a fluorescent acceptor. One of the key requirements for energy transfer is the proximity between the donor and acceptor, with the efficacy of energy transfer being inversely proportional to the sixth power of the distance between donor and acceptor. This, in practice, limits the detection of proximal interactions to <10 nm^4^. BRET is therefore highly suitable for studying protein-protein proximity and/or interactions. Furthermore, the method can be configured to monitor proximity in live cells and in real time^5^. Indeed BRET has been used extensively to study GPCR-protein interactions due to the ability to observe, at least at the population level, the dynamics (i.e. association and dissociation) of the interaction^6^. The main caveat of this technique is that expression of the luminescent donor requires fusion to the GPCR or protein of interest, which is then exogenously expressed^5, 7^. In contrast, the fluorescent acceptor does not need to be a fusion protein and can instead be a fluorophore fused to any number of interacting components (including antibodies or drug-like molecules), as demonstrated for both plasma membrane^8^ and intracellular target engagement^9^. Therefore addressing this exogenous expression requirement for the donor was our priority in this study.

So as to not interfere with the subtleties of the protein interaction, fusion proteins are ideally expressed at near physiological expression levels of the non-fusion native protein^10^. Doing so limits the potential to overwhelm interactions with the endogenous protein population and thus enables investigation of native influences on the interactions of the monitored protein^10–12^. In cell culture models exogenous expression of the donor luciferase fusion proteins is routinely achieved by transient transfection of expression vectors into model cell lines that are easy to transfect and manipulate, but result in a heterologous population of cells with varying levels of over-expressed fusion protein.

Use of cells stably expressing the fusion proteins can overcome the heterologous nature of transient expression models and can allow greater control over the levels of expression^13^. Additionally cells obtained from analogous transgenic animal models expressing luciferase donors and fluorescent acceptors have also been used in BRET assays^14^, however the protein-luciferase fusion in these cases is still exogenously expressed. Furthermore, these model systems can suffer from the continued presence of native protein expressed from the endogenous genome, which may confound the interpretation of protein-protein interactions^10, 11^. Finally, typical model protein expression systems such as cDNA expression plasmids with heterologous promoters do not maintain physiological gene expression and are not subject to the normal endogenous regulators of transcription and translation^15, 16^.

The recent discovery of endonucleases that can be harnessed for site specific DNA cleavage, such as transcription activator-like effector nucleases (TALENs)^17^ and clustered regularly interspaced short palindromic repeats (CRISPR/Cas9 system)^18^, have simplified the modification of endogenous mammalian loci. Fusion of donor luciferases to endogenous proteins via homology-directed repair is now no longer a complex and inefficient exercise^19^. A number of previous studies have used these techniques to knock-in/out^20–22^ or upregulate^23, 24^ GPCRs, or important modulators and regulators of GPCR signalling^25^, as well as to create reporter lines with luciferases or fluorescent proteins^16, 23, 26–28^.

The technical capacity of studying low or near endogenous levels of expression for some proteins can be limited by the sensitivity of detection of the low levels of light generated following substrate oxidation by some of the luciferases traditionally used. Fortunately, the technologies to detect these proteins have similarly advanced, such as the progressive development of BRET configurations that has led to increasingly sensitive BRET assays^12, 29^. The most recent development is the discovery of a novel nanoluciferase (Nluc) from deep sea shrimp *Oplophorus gracilirostris* ^30^. Nluc is a small 19 kD luciferase that emits a bright, stable and spectrally narrow luminescence^30^ and has been used in BRET assays to determine exogenously expressed protein-protein interactions^12, 31^ as well as protein-ligand interactions^8^. This is an advantageous development as the luminescence produced by Nluc has been shown to be 70-fold brighter than that of Rluc8 (a mutant stabilized variant of *Renilla* luciferase)^8^, leading to greater sensitivity at low levels of expression reportedly similar to endogenous levels^12, 30^.

To our knowledge, no study has utilised CRISPR/Cas9 to fuse a luciferase to an endogenous protein and used the resultant genome-edited protein for BRET experiments. To overcome the requirement of BRET assays for the donor luciferase to be exogenously expressed we have used CRISPR/Cas9 mediated homology-directed repair^19^ to insert DNA coding for Nluc into the endogenous genomic loci of *ARRB2* (coding for β-arrestin2) or *CXCR4* (coding for CXC chemokine receptor 4, CXCR4). CXCR4 is a GPCR involved in development, hematopoiesis, organogenesis, and angiogenesis^32^ and is involved in cancer progression and metastasis^33^, immunodeficiency disease encompassed by WHIM syndrome^34^ and acts as a coreceptor in facilitating HIV infection^35^. The resultant fusion proteins acted as sensitive BRET donors from which ligand-induced changes in BRET could be monitored when a fluorescent acceptor was exogenously-expressed. We were able to monitor β-arrestin recruitment, as well as GPCR internalisation and trafficking. This method overcomes the need for ectopic expression of the donor and therefore represents an advance in the current BRET proximity assays.

## Results

### Fusion of Nluc to endogenous CXCR4

A major limitation of BRET is that the technique requires fusion of the luminescent donor to the cellular component of interest, in this case the chemokine GPCR CXCR4. To circumvent this limitation, we used CRISPR/Cas9-meditated homology-directed DNA repair to fuse Nluc to the C-terminus of CXCR4, known to be expressed at low levels (~20 fmol/mg of membrane protein^36^) endogenously in HEK293FT cells. Hemizygous clones were generated and comparison with luminescence from HEK293FT cells transiently transfected with cDNA encoding CXCR4/Nluc showed relatively low levels in HEK293FT cells (Supplementary Fig. 1). Stimulation of cells expressing gene-edited CXCR4/Nluc with CXCL12 resulted in a concentration dependent inhibition of forksolin-mediated cAMP accumulation with nanomolar potency (Supplementary Fig. 1g; pEC50 8.6 ± 0.18) confirming the functionality of CXCR4 following gene editing. We considered that this method of generating receptor fusions is somewhat analogous to the common strategy of generating cell lines with stably integrated expression vectors to increase the sensitivity of transient expression BRET models. However, in these models expression is driven by an exogenous integrated promoter, hyper-methylation of which during continuous culture of these lines can lead to a loss of expression^37^, which genome-edited receptor fusions should not suffer. Indeed, luminescence from expression of the genome-edited CXCR4/Nluc fusion was stable following 20 continuous passages (Supplementary Fig. 1). Furthermore, overexpression of the DNA binding protein YY1, which acts as a repressor of the CXCR4 promoter^38^, resulted in a decrease in observable luminescence in cells expressing the genome-edited CXCR4/Nluc fusion (Supplementary Fig. 1, p < 0.05) without affecting cell number (Supplementary Fig. 1). These results suggest that the addition of Nluc into the CXCR4 locus was not detrimental to the cells and normal endogenous regulators of transcription controlled receptor expression. Additionally, generation time of CRISPR/Cas9-mediated fusions is comparable to that for traditional transgenic stable cell lines^19^.

### Live cell BRET protein-protein interactions using genome-edited CXCR4/Nluc

**Figure 1:**
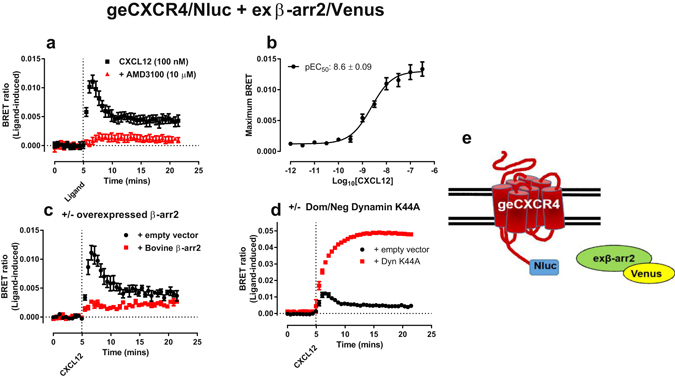
Monitoring β-arrestin2 recruitment to genome-edited CXCR4/Nluc using BRET. HEK293FT cells expressing genome-edited CXCR4 fused to Nluc (geCXCR4/Nluc) transiently transfected with cDNA coding for β-arrestin2/Venus (exβ-arr2/Venus) were used to (**a**) determine ligand-dependent CXCL12 (100 nM) recruitment of β-arrestin2 to CXCR4 in the absence or presence of the CXCR4 antagonist AMD3100 (10 µM). (**b**) Application of CXCL12 (1 pM–300 nM) resulted in a concentration-dependent recruitment of exβ-arr2/Venus to geCXCR4/Nluc. Modulation of CXCL12 (30 nM) mediated exβ-arr2/Venus recruitment to geCXCR4/Nluc by (**c**) additional co-expression of untagged bovine β-arr2 or (**d**) dominant-negative dynamin K44A. (**e**) Schematic representation of the BRET configuration. ‘BRET ratio (ligand-induced)’ was calculated as described in *Methods*. Points represent mean ± S.E.M. of three (c and d), four (**a**) or five (**b**) independent experiments. (**b**) Concentration-response curve fit was by non-linear regression and was used to calculate pEC_50_, with points representing maximum response observed in a kinetic assay.

The newly developed Nluc has previously been used to study protein-protein interactions via BRET in assays with ectopic expression of protein-Nluc fusions in model cell lines^12^, but not with Nluc fused to the endogenous protein. When we transiently expressed β-arrestin2 fused to the fluorescent protein Venus (β-arr2/Venus) in HEK293FT cells expressing genome-edited CXCR4/Nluc, we could monitor the time-course of the ligand-induced increase in BRET in live cells (Fig. 1a). This effect was concentration dependent (Fig. 1b) and was inhibited by the CXCR4 antagonist AMD3100 (10 µM; Fig. 1a), demonstrating that it is the result of CXCL12-specific activation of CXCR4/Nluc. Co-expression of β-arr2/Venus with untagged β-arrestin2 (Fig. 1c) inhibited this response, whereas co-expression of dominant-negative dynamin K44A, which prevents clarithin-coated vesicle formation and thus internalisation, resulted in a substantially more robust BRET signal (Fig. 1d) indicating the specificity of CXCR4/Nluc interactions with β-arr2/Venus in generating the increase in BRET. These results, while showing some differences in kinetic profiles, were in general agreement with those seen when β-arrestin2/Venus was co-expressed with exogenous CXCR4/Nluc (Supplementary Fig. 2) at levels similar to those seen for genome-edited CXCR4 (Supplementary Fig. 1). However, when potency was analysed at the peak of β-arrestin2 recruitment observed in cells expressing genome-edited CXCR4/Nluc fusion receptors we noted a difference in the potency (p < 0.01, two-sided paired t-test, t-value 4.66, df = 7) of CXCL12-mediated β-arr2/Venus recruitment to genome-edited CXCR4/Nluc fusion receptors compared to CXCL12-mediated β-arr2/Venus recruitment to exogenously expressed CXCR4/Nluc (Fig. 1b, Supplementary Fig. 2).

### Live cell BRET protein-protein assays using genome-edited β-arrestin2/Nluc

**Figure 2:**
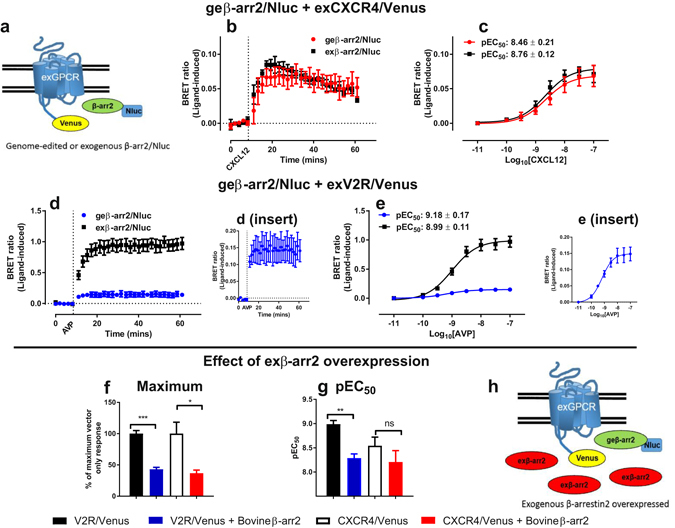
Investigating recruitment of genome-edited β-arrestin2 using BRET. (**a**) Schematic representation of the exogenously expressed GPCR fused to Venus (exGPCR/Venus) and β-arr2/Nluc BRET configuration. HEK293FT cells expressing genome-edited β-arrestin2 fused to Nluc (geβ-arr2/Nluc) transiently transfected with cDNA coding for (**b**,**c**) CXCR4 fused to Venus (exCXCR4/Venus; red circles) or (**d**,**e**) V2R fused to Venus (exV2R/Venus, blue circles) as well as HEK293FT cells transiently co-transfected to express exogenous β-arrestin2 fused to Nluc (exβ-arr2/Nluc, black squares) at near endogenous levels and (**b**,**c**) exCXCR4/Venus or (**d**,**e**) exV2R/Venus. (**b**,**d**) Kinetic profiles of β-arrestin2/Nluc recruitment initiated by addition of CXCL12 (30 nM) or AVP (100 nM) for CXCR4 and V2R respectively. Concentration-dependent recruitment of genome-edited or exogenous β-arrestin2/Nluc to (**c**) exCXCR4/Venus or (**e**) exV2R/Venus mediated by CXCL12 (10 pM–100 nM) or AVP (10 pM–100 nM) respectively. Inserts show geβ-arr2/Nluc recruitment to exV2R/Venus presented in (**d**,**e**). (**f**,**g**) Effect of overexpression of unlabelled exogenous β-arrestin2 (blue and red bars) on maximum recruitment response (**f**) and potency (**g**) in HEK293FT cells expressing geβ-arr2/Nluc transiently transfected with cDNA encoding exCXCR4/Venus (white and red bars) or exV2R/Venus (black and blue bars). (**h**) Schematic representation of the BRET configuration used in (**f**,**g**). ‘BRET ratio (ligand-induced)’ was calculated as described in *Methods*. Points and bars represent mean ± S.E.M. of three or four independent experiments. Statistical analysis by unpaired two-tailed t-test: ns, not significant, t-value = 1.144, df = 6; *p < 0.05, t-value = 3.610, df = 6; **p < 0.01, t-value = 6.105 df = 4; ***p < 0.001, t-value = 9.817 df = 4.

**Figure 3:**
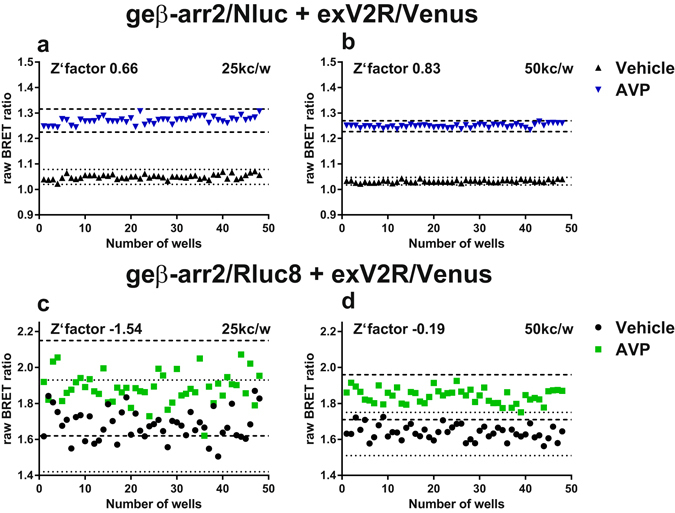
Determining luciferase and assay sensitivity by Z′ factor calculation. HEK293FT cells expressing genome-edited β-arrestin2 fused to Nluc (**a**,**b**, geβ-arr2/Nluc) or Rluc8 (**c**,**d**, geβ-arr2/Rluc8) were transiently transfected with cDNA coding for V2R fused to Venus (exV2R/Venus) and used to calculate Z′ factors. Cells seeded at 25,000 cells/well (25kc/w; **a**,**c**) or 50,000 cells/well (50kc/w; **b**,**d**) were treated with vehicle (black symbols) or AVP (1 µM; blue triangles, green squares). Points represent raw BRET ratio of individual wells ~25 minutes following vehicle or AVP addition. Dashed and dotted lines indicate 3 standard deviations (SD) from the mean of the AVP-treated and vehicle-treated data sets respectively. Z′ factors are calculated as described in *Methods*. Data shown are representative of three independent experiments.

The measurement of β-arrestin2 (also known as arrestin-3) recruitment to GPCRs is of significant interest as it is involved in desensitisation of G protein-mediated signalling, as well as G protein-independent signalling^1^. A number of methods have been used to investigate β-arrestin recruitment, however they have almost exclusively used some form of β-arrestin fused to a reporter protein that is exogenously expressed^5, 39^. BRET has been used extensively to measure β-arrestin recruitment to GPCRs in live cells and has the advantage that it allows for the dynamic measurement of β-arrestin recruitment i.e. association and dissociation of the receptor-β-arrestin complex^5^. Here we investigated genome-edited β-arrestin2 recruitment to exogenously expressed GPCRs using BRET. The luciferases Nluc or Rluc8 were fused to the C-terminus of native β-arrestin2 expressed endogenously in HEK293FT cells using CRISPR/Cas9-meditated homology-directed DNA repair at the *ARRB2* gene. This genome-editing resulted in hemizygous clones from which luminescence could be measured (Supplementary Fig. 3), and which could be used in BRET assays when the acceptor was exogenously expressed. Transient expression of the GPCRs CXCR4, V2R (Fig. 2, Supplementary Fig. 4), β2-adrenoceptor or CCR2 (Supplementary Fig. 5) fused to the fluorescent protein Venus in HEK293FT cells expressing genome-edited β-arr2/luciferase resulted in ligand- and concentration-dependent increases in BRET. Inhibition of this signal by overexpression of untagged β-arrestin indicated that this change in BRET was due to recruitment of genome-edited β-arrestin2/luciferase to the receptor (Fig. 2f and g, Supplementary Fig. 4). Under standard conditions (Fig. 2 and Supplementary Fig. 4) there was no discernible preference as to which luciferase was used, however due to greater brightness it has been reported that Nluc is advantageous at lower levels of protein expression^12^. To ascertain if this was the case in our models, a titration of cell number was performed for lines expressing either genome-edited β-arr2/Nluc or β-arr2/Rluc8 transfected to express exogenous V2R/Venus, thus limiting the observable signal. Under such conditions, Nluc out-performed Rluc8 (Supplementary Fig. 6) with good signals observed down to 5,000 cells/well of a 96-well plate for Nluc, whereas substantial noise was observed below 50,000 cells/well for Rluc8. Furthermore, we used Z′ factor analysis as a quantitative measure of the suitability of the genome-edited β-arr2/luciferase cell lines to be used in high-throughput screening, as well as a proxy for luciferase sensitivity for measuring endogenous protein interactions. In agreement with our previous experiments, Nluc produced superior Z′ factors (Fig. 3; 0.66 [range 0.59 to 0.78] and 0.83 [range 0.80 to 0.87] at 25,000 cells/well and 50,000 cells/well respectively) compared to Rluc8 (Fig. 3; −1.54 [range −1.57 to −1.08] and −0.19 [range −0.66 to −0.14] at 25,000 cells/well and 50,000 cells/well respectively). The consequences of GPCR–β-arrestin2 recruitment are dependent on the stability of the interaction, for example, the tight binding of β-arrestin to V2R is thought to contribute to driving intracellular trafficking towards lysosomal degradation^40^. Furthermore, V2R is reported to not recycle back to the plasma membrane following ligand binding and internalisation^40^. The results reported here monitoring genome-edited β-arrestin2 recruitment support this dogma; V2R/Venus co-expression with genome-edited β-arr2/Nluc or β-arr2/Rluc8 resulted in kinetically stable interactions, which were noticeably different in magnitude when V2R/Venus was co-expressed with exogenous β-arr2/Nluc or β-arr2/Rluc8 (Fig. 2, Supplementary Fig. 4). In contrast, for CXCR4, β_2_-adrenoceptor and CCR2, which bind β-arrestin2 to varying degrees and recycle back to the plasma membrane, the magnitude of genome-edited or exogenous β-arrestin recruitment was similar, with less stable interactions (Fig. 2, Supplementary Figs 4 and 5). Thus V2R/Venus very slowly dissociates from low levels of β-arr2/luciferase in the genome-edited cells, likely effectively sequestering the available β-arr2/luciferase, whereas sufficient reserve in exogenous β-arr2/luciferase lines allows for the generation of a signal of greater magnitude. While exogenous β-arr2/luciferase was transfected to generate levels of luminescence near those observed for native expression (Supplementary Figs 3 and 4), the genome-edited-transient comparison likely over-estimated the endogenous levels due to comparison of a clonal population to a heterologous population. Furthermore, it could not take into account a few highly-expressing cells in the transiently-transfected population potentially generating a disproportionate amount of the BRET response. This indicates that the level of native expression in cells can be difficult to replicate with transient transfection models.

Here we observed a distinct transient-then-plateauing temporal kinetic profile of recruitment of β-arrestin2 to genome-edited CXCR4/Nluc (Fig. 1a). This contrasted with the kinetic profiles of BRET configurations using exogenous CXCR4/Nluc (Supplementary Fig. 2) and exogenous or genome-edited β-arrrestin2/luciferase (Fig. 2) that were much less transient in nature. This difference was independent of the BRET acceptor species used, as substitution of the Venus fluorophore with HaloTag fused to β-arrrestin2 had no observable effect on the endogenous versus exogenous temporal kinetic profiles observed (Supplementary Fig. 7). Unsurprisingly, further analysis of the potency of CXCL12-meditated recruitment of β-arrestin2 to CXCR4 (pEC_50_; Supplementary Fig. 8) revealed that potency was dependent on the BRET configuration used, as noted above, as well as the temporal endpoint selected. When assay configuration and temporal potency differences were combined, we observed 100-fold range of potency values (pEC_50_ 7.3–9.3). Reflecting disparities in receptor reserve, β-arrestin2 expression levels and BRET efficiencies, configurations using genome-edited CXCR4 or β-arrestin2 that have the greatest differences in expression between receptor and β-arrestin2 showed the greatest variance in the potency of CXCL12-mediated β-arrestin2 recruitment. In contrast, smaller changes in potency of CXCL12 were observed in configurations where both CXCR4 and β-arrestin2 were expressed exogenously.

### Internalisation and trafficking of genome-edited CXCR4

**Figure 4:**
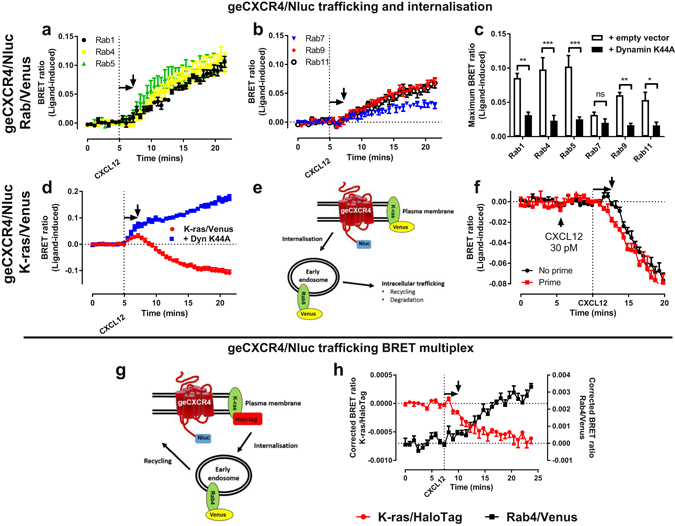
Kinetic profiling of the internalisation and trafficking of genome-edited CXCR4. HEK293FT cells expressing genome-edited CXCR4 fused to Nluc (geCXCR4/Nluc) transiently transfected with cDNA encoding one of the subcellular markers (**a**,**c**) Rab 1, 4 and 5 (**b**,**c**) Rab 7, 9, and 11, or the plasma membrane marker K-ras (**d**) fused to Venus in the absence (**a**–**d**) or presence (**c**,**d**) of co-expressed dominant-negative dynamin K44A. Internalisation and trafficking was induced by CXCL12 (30 nM) added when indicated. (**e**) Schematic representation of the NanoBRET configuration used in the trafficking assay. (**f**) HEK293FT cells expressing genome-edited CXCR4 fused to Nluc (geCXCR4/Nluc) transiently transfected with cDNA coding for Venus/K-ras were used to determine the effect of receptor priming by CXCL12 (30 pM) on internalisation induced by CXCL12 (30 nM). (**g**) Schematic representation of the NanoBRET multiplex configuration. (**h**) HEK293FT cells expressing genome-edited CXCR4 fused to Nluc (geCXCR4/Nluc) transiently co-transfected with cDNA coding for HaloTag/K-ras and Rab4/Venus were used to study genome-edited CXCR4/Nluc receptor internalisation and trafficking induced by CXCL12 (30 nM) in the same cell using a BRET multiplex assay. ‘BRET ratio (ligand-induced)’ was calculated as described in *Methods*. Arrows indicate the delay in internalisation following ligand addition. Points represent mean ± S.E.M. of three (**a**,**b**), four (**h**) or five (**d**,**f**) independent experiments. Bars (**c**) represent maximum response ± S.E.M. of three independent experiments observed in kinetic time-courses, with empty vector controls obtained from (**a**,**b**). Statistical analysis by a two-way ANOVA with a Sidak’s post-test for multiple comparisons. ns, not significant; *p < 0.05; **p < 0.01; ***p < 0.001.

BRET assays have been developed based on bystander BRET interactions to monitor trafficking and internalisation properties of GPCRs by observing changes in proximity to Rab GTPase which are localised to different intracellular compartments^41, 42^. We aimed to investigate if this trafficking assay could be used in cells expressing genome-edited CXCR4/Nluc. Following CXCL12 addition we noted a ligand-dependent increase in BRET and therefore an increase in proximity between exogenously transfected Rab GTPase proteins fused to Venus (Fig. 4a, Rab 1 (biosynthetic endoplasmic reticulum and Golgi) or Rabs 4 or 5 (early endosomes); Fig. 4b, Rabs 7 or 9 (late endosomes/lysosomes) or Rab 11 (transferrin-bearing recycling endosomes)), indicating trafficking to these compartments. Additionally, when the plasma membrane marker K-ras (fused to Venus) was transfected (Fig. 4d), we noted a slight CXCL12-dependent increase in BRET followed by a subsequent decrease. Inhibition of receptor internalisation by overexpression of the dominant-negative dynamin K44A (Fig. 4c and d) resulted in a decrease in ligand-induced internalisation and an increase in receptor at the plasma membrane. These data suggest that following receptor activation, our genome-edited model CXCR4 initially translocates to and/or within the plasma membrane, resulting in accumulation, possibly in structured plasma membrane domains from where signalling and internalisation then occurs. This would be consistent with CXCR4 signalling from lipid-rafts, as well as *in vivo* findings that priming of CXCR4 with low doses of agonist results in increased responsiveness by mediating accumulation in lipid rafts^43, 44^. Furthermore, this would also be consistent with signalling being enhanced following a decrease in receptor mobility at the plasma membrane, due to immobilisation by clathrin-dependent structures following ligand binding^45^. We therefore hypothesised that if low concentration priming results in pre-assembly in lipid rafts, there would be reduction in the delay of internalisation in our model. Priming of genome-edited CXCR4/Nluc using a concentration of CXCL12 sufficient for G protein activation, but not for observable internalisation (30 pM; Fig. 4f), resulted in immediate internalisation following subsequent stimulation with a maximal dose of CXCL12. Due to the robust results we observed in the trafficking assay using the genome-edited CXCR4/Nluc we configured a BRET assay to determine if we could observe internalisation away from the plasma membrane and accumulation in early endosomes in the same cells (Fig. 4g). The NanoBRET multiplexing assay exploits the enhanced luminescence of the donor Nluc and the consequent ability to achieve clear acceptor species spectral separation, using Venus (excitation maximum of 515 nm and emission maximum of 528 nm) and HaloTag in combination with the NCT ligand (excitation maximum of 595 nm and emission maximum of 635 nm)^12^. Cells with genome-edited CXCR4/Nluc were transfected to co-express K-ras/HaloTag and Rab4/Venus, from which we observed internalisation and trafficking to early endosomes following CXCL12 addition (Fig. 3h).

### GPCR Heteromer Investigation Technology (GPCR-HIT) using genome-edited CXCR4/Nluc

**Figure 5:**
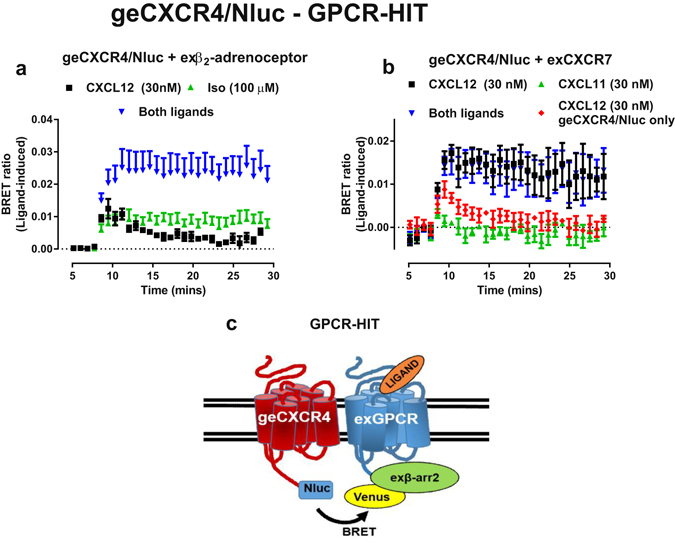
Demonstration of GPCR-HIT BRET assay using genome-edited CXCR4. HEK293FT cells expressing genome-edited CXCR4 fused to Nluc (geCXCR4/Nluc) transiently transfected with cDNA coding for β-arrestin2/Venus and (**a**) β_2_-adrenoceptors or (**b**) CXCR7 were used to carry out BRET assays using the GPCR-HIT configuration. (**a**) Cells were stimulated with CXCL12 (30 nM, black squares), isoprenaline (100 µM, green upward triangles) or both ligands simultaneously (blue downward triangles) or (**b**) CXCL12 (30 nM, black squares), the CXCR7-specific ligand CXCL11 (30 nM, green upward triangles) or both ligands simultaneously (blue downward triangles). (**b**) For comparison, responses mediated by CXCL12 (30 nM, red diamonds) in HEK293FT cells expressing genome-edited CXCR4 fused to Nluc (geCXCR4/Nluc) transiently transfected with only cDNA coding for β-arrestin2/Venus is shown. (**c**) Schematic representation of the GPCR-HIT configuration using NanoBRET. ‘BRET ratio (ligand-induced)’ was calculated as described in *Methods*. Points represent mean BRET ratio ± S.E.M.; bars represent maximum corrected BRET ratio ± S.E.M. observed in kinetic time courses. Ligand added at approximately 8 minutes. Data generated from three independent experiments.

GPCR-HIT has been used to investigate functional receptor interactions^46^. We therefore assessed whether genome-edited CXCR4/Nluc could be used for this assay, using two GPCR combinations previously published as being heteromers, namely β_2_-adrenoceptor-CXCR4^47^ and CXCR4-CXCR7^48, 49^. Using the assay, we observed isoprenaline-mediated recruitment of β-arrestin2/Venus proximal to genome-edited CXCR4/Nluc when β_2_-adrenoceptor and β-arrestin2/Venus were co-expressed with genome-edited CXCR4/Nluc (Fig. 5a). We also noted that co-treatment with both isoprenaline and CXCL12 resulted in a greater than additive increase in ligand-induced BRET ratio. In contrast, no HIT response was observed for Vasopressin 1b receptor (V1bR) even though similar β-arrestin2 translocation to the plasma membrane was observed upon activation of this receptor (Supplementary Fig. 9). When CXCR7 was co-expressed with genome-edited CXCR4/Nluc, a barely discernible and transient BRET response was observed when cells were stimulated with the CXCR7-specific ligand CXCL11. However, we noted a transient-to-stable switch in the temporal β-arrestin2 recruitment profile when cells where stimulated with CXCL12 (which binds both CXCR4 and CXCR7; Fig. 5b), which is consistent with reported observations of prolonged β-arrestin2-mediated signalling by CXCR4-CXCR7 heteromers^48, 49^. This effect is striking when compared to CXCL12 treatment of cells expressing genome-edited CXCR4/Nluc and exogenous β-arrestin2/Venus in the absence of CXCR7 co-transfection.

## Discussion

Here for the first time we have demonstrated that ligand-induced changes in protein proximity can be monitored by BRET when the donor luciferase is genetically fused to a natively expressed protein via CRISPR/Cas9-mediated homology-directed repair and the acceptor species is exogenously expressed. More specifically, this method has been used to study proximity to genome-edited CXCR4 or β-arrestin2 and overcomes the need for ectopic expression of the donor luciferase fusion protein when carrying out BRET assays. This has been exemplified using β-arrestin recruitment and trafficking BRET assays.

For these studies we used a model HEK293 cell line due to its relative ease of manipulation, and like many immortalised cell lines, HEK293 cells have a non-diploid karyotype. The cell lines we generated were hemizyogous for the protein/luciferase fusion, yet even so, in all clones tested these cells generated sufficient luminescence to be used in BRET assays when the acceptor species was exogenously expressed (Supplementary Figs 10 and 11). This approach may be useful for monitoring interactions with proteins expressed at even lower levels if homozygosity is achieved. It should also be noted that without generation of homozygous clones, responses observed in the cells generated here will still have the potential to be influenced by the presence of untagged protein.

The cellular environment can influence protein signalling as well as localisation/compartmentalisation, and will differ between cell types, thus protein-protein interactions are likely the sum of the cellular components. Ideally protein-protein interaction studies would be performed in cell types relevant to their function rather than model cell lines as used here, however CRISPR/Cas9 homology-directed repair is most efficient in dividing cells, with non-dividing cells more likely to undergo non-homologous end joining^50^. Thus homology-directed repair in primary cells with limited culture life or non-dividing cells is challenging^51^. However, investigating interactions with a luciferase-fused protein of interest in such models could conceivably be achieved by: CRISPR/Cas9-mediated generation of knock-in pluripotent stem cells^52^ that could be further differentiated to study interactions in the cell type of interest; using cells obtained from, or the use of, knock-in animals^28^; or engineering existing pathophysiological models. Additionally, non-Cas9, CRISPR/Cpf1 mediated genome engineering has been reported that has the potential to allow for insertions in non-dividing cells^53^.

While we have shown that CRISPR/Cas9-mediated genome engineering overcomes the need for exogenous expression of the donor luciferase, in the model presented the acceptor species still requires exogenous expression, and this will influence the cellular environment. Similarly the levels of acceptor expression will affect efficiency of BRET and therefore the signal observed. The cellular environment will also influence the protein-protein interaction being observed, for example in the case of β-arrestin2 recruitment, β-arrestin1 (as well as untagged β-arrestin2 in the hemizygous lines) will still be present in the cells and interact with the activated receptor. Indeed, as would be expected, in β-arrestin2 recruitment studies we observed differing kinetic and pharmacological profiles depending on exogenous versus genome-edited donor expression and the BRET assay configuration. Without additional investigation it is not possible to draw firm conclusions as to which configuration profile provides a more physiologically-accurate representation of the CXCL12-mediated β-arrestin2 recruitment to CXCR4 in HEK293 cells. Interestingly however, based on the kinetic profiles observed with the genome-edited CXCR4/Nluc recruiting β-arrestin2 and the trafficking assay, where peak β-arrestin2 recruitment aligns with the initiation of internalisation, there are hints at internalisation-driven dissociation of β-arrestin2 from a limited pool of CXCR4/Nluc, which is not observed in the other configurations. These experiments highlight that care should be taken when comparing the pharmacology observed with different BRET assay configurations or cell types.

Given the versatility of CRISPR/Cas9 genome engineering, future studies could aim to substitute Nluc with its self-complementing split-derivative^54^, or use Nluc with fluorescent acceptors such as Venus and/or HaloTag with its NCT ligand fused to more than one endogenous protein in a complementation or BRET assay. Additionally, engineering the genomic loci to express G proteins or β-arrestins with BRET conformational biosensors used previously in overexpressed systems^3, 55^ would allow activation of native untagged proteins to be monitored with reduced impact.

The genome-edited cells do have an advantage in that expression is still controlled by normal regulators of transcription and translation. Furthermore, unlike exogenously-expressed fusion proteins, expression is driven by an endogenous promoter and the endogenous 5′ and 3′ UTRs are still present. In the assays performed here the function of the protein-fusion did not appear to be grossly affected, however any subtle changes to function or changes in expression were not directly investigated. While generally well tolerated, creation of a fusion protein has the potential to affect function and expression levels due to effects on transcription/translation compared to an untagged endogenous protein. Thus the fusion-species should be considered a novel entity, regardless of whether or not the protein is exogenously expressed or is a genome-edited fusion protein under endogenous promotion.

Endogenous proteins are likely to be expressed at lower levels than those achieved in transfection models, particularly with promoters such as CMV and SV40. As such the choice of luciferase used in genome-edited lines requires some consideration. While we find that both luciferases can be used in BRET assays when fused to genome-edited proteins and when the acceptor is exogenously expressed, the Z′ factor analysis indicates that the genome-edited β-arrestin2/Nluc line results in superior reproducibility and sensitivity compared to the β-arrestin2/Rluc8 line. This is presumably largely as a consequence of increased brightness, although given the homozygosity of the clones we cannot rule-out additional allelic recombination in the β-arrestin2/Nluc clone. Furthermore, optimising the donor-acceptor pairing for increased BRET efficiency may increase the sensitivity of Rluc8 at these low levels of expression. Nluc is 70-fold brighter than Rluc8^8^ and it has been reported that this is advantageous for studying proteins expressed at low/near endogenous expression levels^12^, which we can confirm here.

The use of bystander BRET to monitor ligand-induced trafficking to different cellular compartments has been described previously for transiently expressed GPCRs and compartment markers^41, 42, 56–58^. We have now shown that trafficking of genome-edited CXCR4/Nluc can also be observed using this approach. Building a picture of trafficking in this manner assumes that the expressed Rab proteins do not unduly alter GPCR function, and it should be noted that direct Rab-GPCR interactions have been shown for some family A GPCRs^59–62^. This assay generally requires comparison between multiple parallel experiments utilising each individual compartment marker. However, we have also demonstrated that genome-edited proteins can be used for BRET multiplexing as the enhanced quantum yield of Nluc enables simultaneous resonance energy transfer to spectrally separable acceptors (Venus and HaloTag with NCT ligand). Multiple scenarios exist were this approach can be exploited, including the previously described multiplexing of Gα_i_ or Gα_q_ activation with cAMP production^63^.

The GPCR-HIT assay configuration is designed to monitor functional interactions between different GPCRs, as distinct from monitoring homomers that are also likely to be present in the cell^46^. Using genome-edited CXCR4/Nluc in the GPCR-HIT BRET assay, we observed changes in the recruitment profiles of β-arrestin2/Venus consistent with those seen previously using the approach. Heteromerisation of CXCR4 with CXCR7 or the β2-adrenoceptor appears to result in altered receptor pharmacology, in particular with respect to G protein signalling and arrestin recruitment^48, 49^. Furthermore, the heteromers have been implicated in the regulation of various physiological processes, such as T lymphocyte chemotaxis^48^ or breast cancer cellular migration^49^ for CXCR4-CXCR7 and cardiac myocyte contractility and myocyte survival in heart disease progression^47^ for β2-adrenoceptor-CXCR4.

As in this study and previously we and others have shown that the bystander BRET assay can be used to monitor receptor trafficking, it is appropriate to consider the implications of this with regard to the GPCR-HIT assay. We have shown that activation of β2-adrenoceptor, CXCR7 or Vasopressin 1b receptor results in robust recruitment of β-arrestin2/Rluc8 to the plasma membrane and therefore closer proximity to the K-ras/Venus marker. This resulted in a similar magnitude of BRET signal due to energy transfer between Rluc8 and Venus for all three receptors (Supplementary Fig. 9). Given these findings, if CXCR4/Nluc also routinely acts as a non-specific plasma membrane marker, we would expect that translocation of β-arrestin2/Venus to the plasma membrane upon activation of untagged Vasopressin 1b receptor would result in a ligand-induced BRET signal (ie. a GPCR-HIT signal) as seen with β2-adrenoceptor and CXCR7 (Fig. 5), however, this is not the case (Supplementary Fig. 9a).

In conclusion, the aim of this study was to overcome the need to over-express the donor-fused protein and to use this to monitor protein proximity of interest to GPCR pharmacology. There are many GPCR-protein interactions currently monitored by BRET using exogenously expressed proteins, as quantitative real-time investigation of interactions with the endogenous protein in live cells is a demanding and non-trivial exercise. We therefore predict that the use of CRISPR/Cas9 to generate genome-edited proteins fused to luciferases used as BRET donors will have broad application for real-time, live cell molecular pharmacology profiling.

## Methods

### CRISPR/Cas9 mediated targeting of CXCR4 and ARRB2 for homology-directed repair fusion to BRET component proteins

HEK293FT cells expressing genome-edited CXCR4 C-terminally tagged with Nluc, or genome-edited β-arrestin2 C-terminally tagged with Nluc or Rluc8, were generated by CRISPR/Cas9 mediated homology-directed repair as described previously^19^. Briefly, targeting sgRNAs (Supplementary Table 1) were designed for human *CXCR4* and *ARRB2* using the CRISPR Design Tool^64^ (http://crispr.mit.edu/) synthesised (Sigma-Aldrich) and cloned into the pSpCas9(BB)-2A-Puro (PX459) expression construct (from Feng Zhang, Addgene plasmid # 48139)^19^. Templates for homology-directed repair (Supplementary Tables 2 and 3) were designed using the UCSC genome browser (http://genome.ucsc.edu/, Human genome assembly (GRCh38/hg38) December 2013^65^) and synthesised by GeneArt (Thermo Fisher Scientific) consisting of homology arms surrounding linker-XbaI-*seq*-XhoI-ApaI to allow for the sub-cloning of different luciferases (Supplementary Table 4). Sequences were verified by Sanger sequencing by the Australian Genome Research Facility (AGRF, Perth).

sgRNA cloned into the px459 expression construct and the corresponding repair template were transfected into HEK293FT cells seeded in 24-well plates maintained at 37 °C in 5% CO_2_ and complete media (Dulbecco’s modified Eagle’s medium (DMEM) containing 0.3 mg/ml glutamine, 100 IU/ml penicillin and 100 μg/ml streptomycin (Thermo Fisher Scientific, Scoresby, VIC, Australia) supplemented with 10% foetal bovine serum (FBS, Bovogen) and 400 μg/ml geneticin (Thermo Fisher Scientific), using Fugene 6 (Promega) 24 h following seeding with 100,000 cells/well. Cells were cultured for 24 h then treated with puromycin (0.3 ug/ml, Thermo Fisher Scientific) for 3 days to select for transfected cells. Following selection, cells were cultured without puromyocin for 1 day then seeded in white 96-well plates (Grenier) at 1 cell per well and allowed to expand for 2–3 weeks. Single colonies were screened for luminescence following the addition of furimazine (5 µM), or coelenterazine h (1 µM) (Promega) using a VICTOR Light plate reader with Wallac 1420 software (PerkinElmer). Positive clones were seeded in 6-well plates and expanded before cells were collected for genotyping by PCR and Sanger sequencing. Multiple hemizygous clones of each genome-edited protein were initially tested for functionality. We tested 3 clones for genome-edited CXCR4/Nluc and β-arrestin2/Nluc and 2 for β-arrestin2/Rluc8. Each clone displayed similar results in the assays performed (Supplementary Figs 10 and 11). A single clone of each type was selected for further testing: clone H7 of CXCR4/Nluc, clone F1 of ARRB2/Nluc and clone B3 of ARRB2/Rluc8. Except for Supplementary Figs 10 and 11, all data shown were generated using these clones, including Sanger sequencing and PCR bands.

### Sequencing of the genomic loci

Genomic DNA was extracted from positive HEK293FT clones using a Zymo gDNA extraction kit (Integrated Sciences, Chatswood, NSW, Australia) and amplified by PCR (see primers in Supplementary Figs 1 and 3, and Supplementary Table 5) using KOD Hot start polymerase (Merck Millipore, Bayswater, VIC, Australia) under standard conditions. PCR products were separated by gel electrophoresis then extracted and purified using a gel extraction kit (Qiagen, Chadstone, VIC, Australia). In-frame insertion of the luciferase was confirmed by Sanger sequencing (AGRF) (Supplementary Figs 1, 3 and 4).

### Cell culture and transfection

Wildtype or CRISPR/Cas9-modified HEK293FT cells, maintained as described above, were transiently transfected according to the manufacturer’s instructions using Fugene 6 transfection reagent (Promega) with exogenous cDNA 24 h after seeding 350,000–400,000 cells/well in a 6-well plate. Cells were harvested with 0.05% Trypsin-EDTA (Thermo Fisher Scientific) and seeded into poly-L-lysine coated white 96-well plates at 50,000–100,000 cells/well in phenol-red free DMEM containing 25 mM HEPES, 0.3 mg/ml glutamine, 100 IU/ml penicillin and 100 μg/ml streptomycin supplemented with 5–10% FBS 24 h before performing an assay.

### Analysis of genome-edited CXCR4/Nluc recruiting β-arrestin2/Venus

CRISPR/Cas9-modified HEK293FT cells expressing genome-edited CXCR4/Nluc were transiently transfected with cDNA encoding β-arrestin2/Venus. In parallel, wildtype HEK293FT cells were transiently transfected with cDNA coding for CXCR4/Nluc to generate near endogenous levels of expression (Supplementary Fig. 1, 25 ng per well of a 6 well plate) as well as β-arrestin2/Venus. 24 h post-transfection, cells were harvested into white 96-well plates at 80,000–100,000 cells/well in phenol-red free DMEM. 48 h post-transfection, cells were serum starved for 30 min at 37 °C in 5% CO_2_ then incubated with furimazine (5 µM) in Hanks buffered saline solution without calcium or magnesium (HBSS, Thermo Fisher Scientific) for 5 min before real-time BRET measurements were taken at 37 °C using a CLARIOstar multi-label plate reader (BMG Labtech, Mornington, VIC, Australia). Filtered light emissions were sequentially measured at 420–480 nm and 520–620 nm. The BRET signal was calculated by subtracting the ratio of the acceptor 520–620 nm emission over the donor 420–480 nm emission for a vehicle-treated cell sample from the same ratio for a sample treated with agonist. In this calculation, the vehicle-treated cell sample represents the background, eliminating the requirement for measuring a donor-only control sample^7^. Following the establishment of a baseline, cells were treated with HBSS with or without CXCL12. In subsets of experiments the following modifications were made: a) the effect of CXCL12 was measured in the absence or presence of AMD3100 (10 µM); b) additional cDNA encoding the dominant-negative dynamin mutant K44A, bovine β-arrestin2 or the equivalent empty vector was co-transfected into cells; c) cDNA encoding CXCR4 fused to Nluc transfected into wildtype HEK293FT cells was titrated with cells seeded at 50,000 cells/well in parallel with cells expressing genome-edited CXCR4/Nluc.

### Analysis of genome-edited CXCR4/Nluc recruiting β-arrestin2/HaloTag

Analysis was performed as per the method described above for recruitment of β-arrestin2/Venus, with the following modifications: CRISPR/Cas9-modified HEK293FT cells expressing genome-edited CXCR4/Nluc were transiently transfected with cDNA encoding β-arrestin2/HaloTag. In parallel, unmodified HEK293FT cells were transiently transfected with cDNA encoding CXCR4/Nluc as well as β-arrestin2/HaloTag. 48 h post-transfection, the media was aspirated and cells were incubated with HaloTag NCT ligand (0.25 µM final concentration) in HBSS for 1 h at 37 °C in 5% CO_2_ to which furimazine (6.6 µM final concentration) was added and incubated for a further 5 min before real-time BRET measurements were taken at 37 °C using a CLARIOstar multi-label plate reader. Filtered light emissions were sequentially measured at 420–480 nm and >610 nm (610LP). The BRET signals were calculated by subtracting the ratio of 610LP over 420–480 nm emission for a vehicle-treated cell sample from the same ratio for a sample treated with agonist.

### Z′ factor analysis

Z′ factor analysis can be used to determine the potential suitability of an assay for high-throughput screening^66^. Briefly, HEK293FT cells expressing genome-edited β-arrestin2/Nluc or Rluc8 were transiently transfected with cDNA encoding V2R/Venus and processed as described above. 48 h post-transfection, cells seeded at 25,000 or 50,000 cells/well were serum starved for 30 min at 37 °C in 5% CO_2_. They were then incubated with furimazine (5 µM for Nluc) or coelenterazine h (1 µM for Rluc8) in HBSS for 5 min before real-time BRET measurements were taken at 37 °C using a LUMIstar plate reader (BMG Labtech). Following the establishment of a baseline, cells in rows A, D, G and H were treated with HBSS and cells in rows B, C, E and F with AVP (1 µM) in HBSS. Filtered light emissions were simultaneously measured at 460–490 nm and 520–550 nm. The raw BRET ratios (520–550 over 460–490 nm) were calculated for both the AVP (positive control) and vehicle-treated (negative control) samples without subtraction of the ratio for the vehicle-treated sample. These values were then used to calculate the Z′ factors using the following equation: Z′ = 1 − [(3 SD of positive control + 3 SD of negative control)/(mean of positive control − mean of negative control)]^66^.

### Internalisation and trafficking

Internalisation and trafficking assays were performed using HEK293FT cells expressing genome-edited CXCR4/Nluc as per the β-arrestin2/Venus recruitment assays, except that subcellular compartment markers fused to the C-terminus of Venus^42^ were substituted for β-arrestin2/Venus in the transfection mix, namely Rab GTPase markers (Rab 1, biosynthetic endoplasmic reticulum and Golgi; Rabs 4 and 5, early endosomal; Rabs 7 and 9, late endosomes/lysosomes; or Rab 11, transferrin-bearing recycling endosomes) or a c-terminal fragment of K-ras acting as a plasma membrane marker. These subcellular compartment markers have been characterized previously^42^. In a subset of experiments, cells were transfected with additional cDNA coding for the dominant-negative dynamin mutant K44A or the equivalent empty vector. Where the effect of a priming dose on CXCR4 internalisation was investigated, following the establishment of a baseline, cells were treated with HBSS with or without CXCL12 (30 pM). BRET was then monitored for a time equivalent to the original baseline then the cells were treated again with HBSS with or without CXCL12 (100 µM final concentration).

### Monitoring CXCR4/Nluc internalisation and trafficking by BRET multiplexing

HEK293FT cells expressing genome-edited CXCR4/Nluc were transiently transfected with cDNA encoding Venus/Rab4 and HaloTag/K-ras. 24 h post-transfection, cells were harvested into white 96-well plates at 80,000–100,000 cells/well in phenol-red free DMEM. 48 h post-transfection, the media was aspirated and cells were incubated with HaloTag NCT ligand (0.25 µM final concentration, Promega) in HBSS for 1 h at 37 °C in 5% CO_2_ to which furimazine (6.6 µM final concentration) was added and incubated for a further 5 min before real-time BRET measurements were taken at 37 °C using a CLARIOstar multi-label plate reader. Filtered light emissions were sequentially measured at 420–480 nm, 520–580 nm and 610–710 nm. For Venus and HaloTag-NCT respectively, the BRET signals were calculated by subtracting the ratio of 520–580 nm emission or 610–710 nm over 420–480 nm emission for a vehicle-treated cell sample from the same ratio for a sample treated with agonist.

### Analysis of genome-edited β-arrestin2-luciferase recruitment to exogenously expressed GPCR

HEK293FT cells expressing genome-edited β-arrestin2/Nluc or β-arrestin2/Rluc8 were transiently transfected with cDNA encoding a GPCR fused to Venus and in parallel wildtype HEK293FT cells were transiently transfected with the same GPCR as well as cDNA encoding β-arrestin2/Nluc or β-arrestin2/Rluc8 to generate near endogenous levels of β-arrestin2 expression (Supplementary Fig. 3; 25 ng per well of a 6 well plate, near but higher to ensure endogenous levels did not overly interfere with the response). 24 h post-transfection, cells were harvested into white 96-well plates at 50,000 or 100,000 cells/well for Nluc and Rluc8 expressing cells respectively in phenol-red free DMEM containing 5% FBS. 48 h post-transfection, cells were serum starved for 1 h at 37 °C in 5% CO_2_ then incubated with furimazine (2.5 µM for Nluc) or coelenterazine h (0.5 µM for Rluc8) in HBSS for 5 min before real-time BRET measurements were taken at 37 °C using a LUMIstar plate reader. Following the establishment of a baseline, cells were treated with HBSS with or without agonist. Filtered light emissions were simultaneously measured at 460–490 nm and 520–550 nm. The BRET signal was calculated as above. In subsets of experiments the following modifications were made: a) cell numbers seeded into 96-well plates were titrated to 5,000 cells/well; b) additional cDNA coding for bovine β-arrestin2 was transfected into cells; c) the cDNA amount encoding β-arrestin2 fused to Nluc or Rluc8 transfected into wildtype HEK293FT cells was titrated.

### GPCR-Heteromer Investigation Technology (GPCR-HIT) assays using genome-edited CXCR4/Nluc

The method for GPCR-HIT has been described previously^46^. Briefly HEK293FT cells expressing genome-edited CXCR4/Nluc were transiently transfected with cDNA encoding β-arrestin2/Venus as well as cDNA encoding an unlabelled GPCR. 24 h post-transfection, cells were harvested into white 96-well plates at 80,000–100,000 cells/well in phenol-red free DMEM. 48 h post-transfection, cells were serum starved for 30 min at 37 °C in 5% CO_2_ then incubated with furimazine (5 µM) in HBSS for 5 min before real-time BRET measurements were taken at 37 °C using a LUMIstar plate reader with the parameters defined above. Following the establishment of a baseline, cells were treated with HBSS with or without CXCL12, ligand for the unlabelled GPCR or CXCL12 and the second ligand. Data were analysed as described previously.

### Effect of YY1 overexpression

HEK293FT cells expressing genome-edited CXCR4/Nluc were transfected with cDNA encoding YY1. 24 h post-transfection, cells were harvested in parallel into white 96-well plates (for luminescence measurements) and black 96-well plates (for fluorescence measurements) at 30,000 cells/well in phenol-red free DMEM containing 5% FBS. 72 h post-transfection, cells for luminescence measurements were serum starved for 30 min at 37 °C in 5% CO_2_ then incubated with furimazine (2.5 µM) in HBSS for 5 min before luminescence was measured through a 450–460 nm filter at 37 °C using a CLARIOstar plate reader. To control for changes in cell number following serum starvation, cells in the black 96 well plates were incubated with Hoescht 33342 (100 µM) in HBSS for 10 min at room temperature then washed three times for 5 min before fluorescence intensity was measured on a CLARIOStar plate reader with excitation at 345–365 nm and emission detection at 440–460 nm.

### Inhibition of cAMP accumulation

cAMP accumulation was measured using a homogenous time-resolved fluorescence (HTRF) cAMP dynamic 2 assay kit (CisBio Bioassays) following the manufacturer’s instructions. Briefly, HEK293FT cells expressing genome-edited CXCR4/Nluc were seeded into white 96-well plates at 20,000 cells per well. After 24 h, cells were treated with forskolin (5 µM) in the absence or presence of increasing concentrations of CXCL12 diluted in stimulation buffer containing 3-isobutyl-1-methylxanthine (0.5 mM) and incubated at 37 °C in 5% CO_2_ for 30 min. Cells were then lysed by addition of the HTRF reagents and incubated for 1 h at room temperature before fluorescence was measured at 620 and 665 nm, 50 μs after excitation at 337 nm using a CLARIOstar plate reader.

### Materials

AMD3100 octahydrochloride hydrate (AMD3100), Arginine Vasopressin (AVP), forskolin, 3-isobutyl-1-methylxanthine and isoprenaline were purchased from Sigma Aldrich (Castle Hill, NSW, Australia), CCL2, CXCL11 and CXCL12, were from Preprotech (supplied by Lonza, Australia) or Proteowa (Murdoch, WA, Australia). HEK293FT cells (Catalogue number R70007) were purchased directly from Thermo Fisher Scientific. Cells were used as a model system to study the molecular pharmacological properties of GPCR signalling. Mycoplasma testing was performed routinely. All cell culture reagents were purchased from Thermo Fisher Scientific, except fetal bovine serum (Bovogen) and the 6-well and white 96-well plates (Greiner), which were purchased from (Interpath Services, VIC, Australia). Coelenterazine h and furimazine, as well as the HaloTag NCT ligand, were from Promega. Preparation or source of cDNA constructs has been described previously: β-arrestin2/Venus^67^ β-arrestin2/Rluc8^68^; CCR2^46^; dynamin K44A^69^; Venus/Rabs 1, 4, 5, 7, 9 and 11^42^; Venus/K-ras^42^; and V2R/Venus^69^. β-arrestin2/Nluc, CCR2/Venus and CXCR4/Venus were sub-cloned from existing constructs using conventional methods. HaloTag/K-ras was synthesized by GeneArt. Untagged constructs encoding β_2_-adrenoceptor, CXCR4, CXCR7 and V1bR were from the Missouri S&T cDNA Resource Center. Bovine β-arrestin2 was kindly provided by Jeffrey Benovic (Thomas Jefferson University). The coding sequence of YY1 (corresponding to RefSeq mRNA: NM_003403.3) was codon optimised for human expression and synthesised as a gBlock® gene fragment by Integrated DNA Technologies (Baulkham Hills, NSW). Additional bases on the 5′ (tgacgtcggtacc) and 3′ (ctcgagttacgta) ends were included to allow for in-frame cohesive end sub-cloning of the YY1 coding sequence into a pcDNA3.1 expression vector using the restriction sites KpnI and XhoI (underlined) and the standard manufacturer’s instructions. Construct sequences were routinely confirmed by Sanger sequencing (AGRF).

### Data Presentation and Statistical Analysis

Assays were performed in technical duplicates or triplicates resulting in each independent experiment as indicated in the text. The n values in the text refer to the number of independent repeat experiments. Data were analysed using GraphPad Prism 6 (GraphPad Software, Inc, CA, USA) with α designated as 0.05. For BRET kinetic assays, time of ligand/vehicle addition is indicated on the x-axis or figure legends. Concentration-response curves were generated using data at maximum response or at a specific time as indicated in the figure legends, with curve fits plotted by non-linear regression analysis performed by GraphPad Prism, which was used to calculate pEC_50_ values. Statistical analysis, indicated in figure legends, was determined using ANOVA with appropriate post-hoc tests for multiple comparisons or unpaired t-test. Where possible, data were checked for normal distribution as well as consistent variance between groups statistically analysed. We have reported previously that based on our experience, a minimum of three repeat experiments provides sufficient power to observe a difference of interest^8^. BRET data were generated using a CLARIOstar or LUMIstar plate reader. As settings were progressively optimised throughout the course of the study, this can result in variations in the magnitude of ligand-induced BRET ratios, but not the kinetics of response. For reference, specific parameters for each experiment are presented in Supplementary Table 6. The data that support the findings of this study, as are the materials used, are available from the corresponding authors upon reasonable request.

## Electronic supplementary material

Supplementary DOC File

## References

[1] Smith JS, Rajagopal S (2016). The beta-Arrestins: Multifunctional Regulators of G Protein-coupled Receptors. J Biol Chem.

[2] Lee MH (2016). The conformational signature of beta-arrestin2 predicts its trafficking and signalling functions. Nature.

[3] Nuber S (2016). beta-Arrestin biosensors reveal a rapid, receptor-dependent activation/deactivation cycle. Nature.

[4] Dacres H, Michie M, Wang J, Pfleger KD, Trowell SC (2012). Effect of enhanced Renilla luciferase and fluorescent protein variants on the Forster distance of Bioluminescence resonance energy transfer (BRET). Biochem Biophys Res Commun.

[5] Pfleger KD, Eidne KA (2006). Illuminating insights into protein-protein interactions using bioluminescence resonance energy transfer (BRET). Nat Methods.

[6] Lohse MJ, Nuber S, Hoffmann C (2012). Fluorescence/bioluminescence resonance energy transfer techniques to study G-protein-coupled receptor activation and signaling. Pharmacol Rev.

[7] Pfleger KD, Seeber RM, Eidne KA (2006). Bioluminescence resonance energy transfer (BRET) for the real-time detection of protein-protein interactions. Nat Protoc.

[8] Stoddart LA (2015). Application of BRET to monitor ligand binding to GPCRs. Nat Methods.

[9] Robers MB (2015). Target engagement and drug residence time can be observed in living cells with BRET. Nature Communications.

[10] Gibson TJ, Seiler M, Veitia RA (2013). The transience of transient overexpression. Nat Methods.

[11] Mahen R (2014). Comparative assessment of fluorescent transgene methods for quantitative imaging in human cells. Mol Biol Cell.

[12] Machleidt T (2015). NanoBRET–A Novel BRET Platform for the Analysis of Protein-Protein Interactions. ACS Chem Biol.

[13] Savage EE, Wootten D, Christopoulos A, Sexton PM, Furness SG (2013). A simple method to generate stable cell lines for the analysis of transient protein-protein interactions. Biotechniques.

[14] Audet M, Lagace M, Silversides DW, Bouvier M (2010). Protein-protein interactions monitored in cells from transgenic mice using bioluminescence resonance energy transfer. FASEB J.

[15] Kain, S. R. & Ganguly, S. Overview of genetic reporter systems. *Curr Protoc Mol Biol* Chapter 9, Unit9 6, doi:10.1002/0471142727.mb0906s36 (2001). 10.1002/0471142727.mb0906s36 18265284

[16] Rojas-Fernandez A (2015). Rapid generation of endogenously driven transcriptional reporters in cells through CRISPR/Cas9. Sci Rep.

[17] Cermak T (2011). Efficient design and assembly of custom TALEN and other TAL effector-based constructs for DNA targeting. Nucleic Acids Res.

[18] Cong L (2013). Multiplex genome engineering using CRISPR/Cas systems. Science.

[19] Ran FA (2013). Genome engineering using the CRISPR-Cas9 system. Nat Protoc.

[20] Hou P (2015). Genome editing of CXCR4 by CRISPR/cas9 confers cells resistant to HIV-1 infection. Sci Rep.

[21] Schumann K (2015). Generation of knock-in primary human T cells using Cas9 ribonucleoproteins. Proc Natl Acad Sci U S A.

[22] Mandal PK (2014). Efficient ablation of genes in human hematopoietic stem and effector cells using CRISPR/Cas9. Cell Stem Cell.

[23] Kamiyama D (2016). Versatile protein tagging in cells with split fluorescent protein. Nat Commun.

[24] Tanenbaum ME, Gilbert LA, Qi LS, Weissman JS, Vale RD (2014). A protein-tagging system for signal amplification in gene expression and fluorescence imaging. Cell.

[25] Alvarez-Curto E (2016). Targeted Elimination of G proteins and Arrestins Defines their Specific Contributions to both Intensity and Duration of G protein-Coupled Receptor Signalling. J Biol Chem.

[26] Ratz M, Testa I, Hell SW, Jakobs S (2015). CRISPR/Cas9-mediated endogenous protein tagging for RESOLFT super-resolution microscopy of living human cells. Sci Rep.

[27] Lackner DH (2015). A generic strategy for CRISPR-Cas9-mediated gene tagging. Nat Commun.

[28] Yang H (2013). One-step generation of mice carrying reporter and conditional alleles by CRISPR/Cas-mediated genome engineering. Cell.

[29] Molinari P, Casella I, Costa T (2008). Functional complementation of high-efficiency resonance energy transfer: a new tool for the study of protein binding interactions in living cells. Biochem J.

[30] Hall MP (2012). Engineered luciferase reporter from a deep sea shrimp utilizing a novel imidazopyrazinone substrate. ACS Chem Biol.

[31] Goyet E, Bouquier N, Ollendorff V, Perroy J (2016). Fast and high resolution single-cell BRET imaging. Sci Rep.

[32] Zou YR, Kottmann AH, Kuroda M, Taniuchi I, Littman DR (1998). Function of the chemokine receptor CXCR4 in haematopoiesis and in cerebellar development. Nature.

[33] Zhao H (2015). CXCR4 over-expression and survival in cancer: a system review and meta-analysis. Oncotarget.

[34] Hernandez PA (2003). Mutations in the chemokine receptor gene CXCR4 are associated with WHIM syndrome, a combined immunodeficiency disease. Nat Genet.

[35] Feng Y, Broder CC, Kennedy PE, Berger EA (1996). HIV-1 entry cofactor: functional cDNA cloning of a seven-transmembrane, G protein-coupled receptor. Science.

[36] Busillo JM (2010). Site-specific phosphorylation of CXCR4 is dynamically regulated by multiple kinases and results in differential modulation of CXCR4 signaling. J Biol Chem.

[37] Pikaart MI, Recillas-Targa F, Felsenfeld G (1998). Loss of transcriptional activity of a transgene is accompanied by DNA methylation and histone deacetylation and is prevented by insulators. Genes & Development.

[38] Moriuchi M, Moriuchi H, Margolis DM, Fauci AS (1999). USF/c-Myc enhances, while Yin-Yang 1 suppresses, the promoter activity of CXCR4, a coreceptor for HIV-1 entry. J Immunol.

[39] Bassoni DL, Raab WJ, Achacoso PL, Loh CY, Wehrman TS (2012). Measurements of beta-arrestin recruitment to activated seven transmembrane receptors using enzyme complementation. Methods Mol Biol.

[40] Shenoy SK, Lefkowitz RJ (2003). Trafficking patterns of beta-arrestin and G protein-coupled receptors determined by the kinetics of beta-arrestin deubiquitination. J Biol Chem.

[41] Lan TH, Kuravi S, Lambert NA (2011). Internalization dissociates beta2-adrenergic receptors. PLoS One.

[42] Tiulpakov A (2016). Mutations of Vasopressin Receptor 2 Including Novel L312S Have Differential Effects on Trafficking. Mol Endocrinol.

[43] Wysoczynski M (2005). Incorporation of CXCR4 into membrane lipid rafts primes homing-related responses of hematopoietic stem/progenitor cells to an SDF-1 gradient. Blood.

[44] Busillo JM, Benovic JL (2007). Regulation of CXCR4 signaling. Biochim Biophys Acta.

[45] Beletkaia E (2016). CXCR4 signaling is controlled by immobilization at the plasma membrane. Biochim Biophys Acta.

[46] See HB, Seeber RM, Kocan M, Eidne KA, Pfleger KD (2011). Application of G protein-coupled receptor-heteromer identification technology to monitor beta-arrestin recruitment to G protein-coupled receptor heteromers. Assay Drug Dev Technol.

[47] LaRocca TJ (2010). beta2-Adrenergic receptor signaling in the cardiac myocyte is modulated by interactions with CXCR4. J Cardiovasc Pharmacol.

[48] Levoye A, Balabanian K, Baleux F, Bachelerie F, Lagane B (2009). CXCR7 heterodimerizes with CXCR4 and regulates CXCL12-mediated G protein signaling. Blood.

[49] Decaillot FM (2011). CXCR7/CXCR4 heterodimer constitutively recruits beta-arrestin to enhance cell migration. J Biol Chem.

[50] Iyama T, Wilson DM (2013). DNA repair mechanisms in dividing and non-dividing cells. DNA Repair (Amst).

[51] Chan F, Hauswirth WW, Wensel TG, Wilson JH (2011). Efficient mutagenesis of the rhodopsin gene in rod photoreceptor neurons in mice. Nucleic Acids Res.

[52] Merkle FT (2015). Efficient CRISPR-Cas9-mediated generation of knockin human pluripotent stem cells lacking undesired mutations at the targeted locus. Cell Rep.

[53] Zetsche B (2015). Cpf1 is a single RNA-guided endonuclease of a class 2 CRISPR-Cas system. Cell.

[54] Zhao J, Nelson TJ, Vu Q, Truong T, Stains CI (2016). Self-Assembling NanoLuc Luciferase Fragments as Probes for Protein Aggregation in Living Cells. ACS Chem Biol.

[55] Irannejad R (2013). Conformational biosensors reveal GPCR signalling from endosomes. Nature.

[56] Jensen DD (2013). The bile acid receptor TGR5 does not interact with beta-arrestins or traffic to endosomes but transmits sustained signals from plasma membrane rafts. J Biol Chem.

[57] Lan TH, Liu Q, Li C, Wu G, Lambert NA (2012). Sensitive and high resolution localization and tracking of membrane proteins in live cells with BRET. Traffic.

[58] Namkung, Y. *et al*. Monitoring G protein-coupled receptor and [beta]-arrestin trafficking in live cells using enhanced bystander BRET. *Nat Commun* **7**, doi:10.1038/ncomms12178 (2016). 10.1038/ncomms12178 PMC4942582 27397672

[59] Esseltine JL, Dale LB, Ferguson SS (2011). Rab GTPases bind at a common site within the angiotensin II type I receptor carboxyl-terminal tail: evidence that Rab4 regulates receptor phosphorylation, desensitization, and resensitization. Mol Pharmacol.

[60] Esseltine JL, Ferguson SS (2013). Regulation of G protein-coupled receptor trafficking and signaling by Rab GTPases. Small GTPases.

[61] Dale LB, Seachrist JL, Babwah AV, Ferguson SS (2004). Regulation of angiotensin II type 1A receptor intracellular retention, degradation, and recycling by Rab5, Rab7, and Rab11 GTPases. J Biol Chem.

[62] Seachrist JL, Anborgh PH, Ferguson S (2000). S. beta 2-adrenergic receptor internalization, endosomal sorting, and plasma membrane recycling are regulated by rab GTPases. J Biol Chem.

[63] Breton B (2010). Multiplexing of multicolor bioluminescence resonance energy transfer. Biophys J.

[64] Hsu PD (2013). DNA targeting specificity of RNA-guided Cas9 nucleases. Nat Biotechnol.

[65] Kent WJ (2002). The human genome browser at UCSC. Genome Res.

[66] Zhang JH, Chung TD, Oldenburg KR (1999). A Simple Statistical Parameter for Use in Evaluation and Validation of High Throughput Screening Assays. J Biomol Screen.

[67] Kocan M, See HB, Seeber RM, Eidne KA, Pfleger KD (2008). Demonstration of improvements to the bioluminescence resonance energy transfer (BRET) technology for the monitoring of G protein-coupled receptors in live cells. J Biomol Screen.

[68] Porrello ER (2011). Heteromerization of angiotensin receptors changes trafficking and arrestin recruitment profiles. Cell Signal.

[69] Kocan M (2009). Agonist-independent interactions between beta-arrestins and mutant vasopressin type II receptors associated with nephrogenic syndrome of inappropriate antidiuresis. Mol Endocrinol.

