# Supplementary material for: Using nanoBRET and CRISPR/Cas9 to monitor proximity to a genome-edited protein in real-time
Source: Sci Rep. 2017 Jun 9;7:3187. doi: 10.1038/s41598-017-03486-2 (PMC5466623; doi:10.1038/s41598-017-03486-2)
Supplement: Supplementary file 1 — Supplementary DOC File [file 41598_2017_3486_MOESM1_ESM.doc]

**Using nanoBRET and CRISPR/Cas9 to monitor proximity to a genome-edited protein in real-time.**

Carl W White, Hannah K Vanyai, Heng B See, Elizabeth KM Johnstone, and Kevin D G Pfleger

**
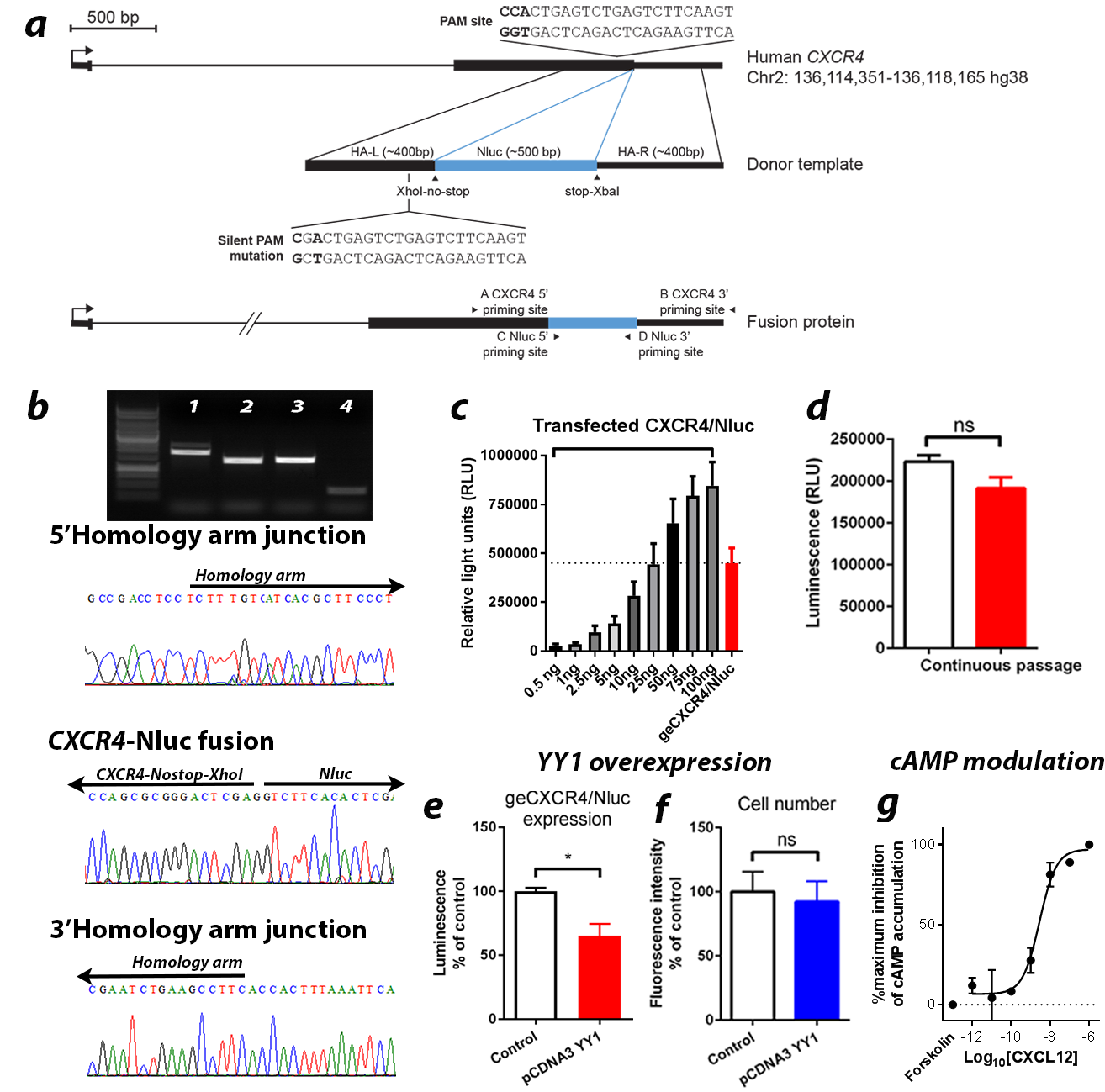
**

**Supplementary Figure 1: Generation of HEK293FT cells expressing genome-edited CXCR4/Nluc. (a)** Schematic representation of targeting the genome to fuse Nluc DNA to the end of *CXCR4* using CRISPR/Cas9-mediated homology-directed DNA repair. HA-R and HA-L are the right and left arms of the homology repair template respectively. Horizontal arrows indicate approximate primer binding sites: A and B, CXCR4 forward and reverse priming sites; C and D, Nluc forward and reverse priming sites. (**b**) Gel electrophoresis and sequencing of PCR products generated using the forward and reverse primers; A-B, lane 1 showing bands for both the wildtype and insert, A-D lane 2, C-B lane 3 and C-D lane 4 by amplification of genomic DNA extracted from HEK293FT cells engineered to express genome-edited CXCR4/Nluc, indicating the presence of hemizygous homologous recombination at the site of interest. (**c**) Comparison of luminescence generated by HEK293FT cells transiently transfected with cDNA coding for CXCR4/Nluc with luminescence generated by HEK293FT cells expressing genome-edited CXCR4/Nluc. (**d**) Comparison of luminescence generated by HEK293FT cells expressing genome-edited CXCR4/Nluc before (white bars) or after (red bars) continuous culture for 20 passages. **(e** and **f)** HEK293FT cells expressing CXCR4/Nluc were transfected with (red and blue bars) or without (white bars) cDNA encoding the DNA binding protein YY1. **(e)** Luminescence as a proxy for CXCR4/Nluc expression measured 72 hours following transfection and **(f)** fluorescence intensity observed in wells stained with Hoescht 33342 (100 µM). **(g)** Inhibition of forskolin (5 µM) mediated cAMP accumulation by CXCL12 (1pM - 1 µM) in HEK293FT cells expressing genome-edited CXCR4/Nluc. Statistical analysis by paired (**e**, t-value=4.696, df=2 and **f**, t-value=1.284 df=2) or unpaired (**d**, t-value=2.157 df=4) t-test. ns, not significant; *, p<0.05. Bars or points represent **(c-d)** mean relative light units (RLU) ± S.E.M. or **(e-f)** % of control or **(g)** % of maximum inhibition of forskolin-mediated cAMP accumulation ± S.E.M. of five (c) or three (d-g) independent experiments. N.B. RLU obtained in (**d**) were measured in parallel using different gain parameters to those in (**c**) (See Supplementary table 6).

**
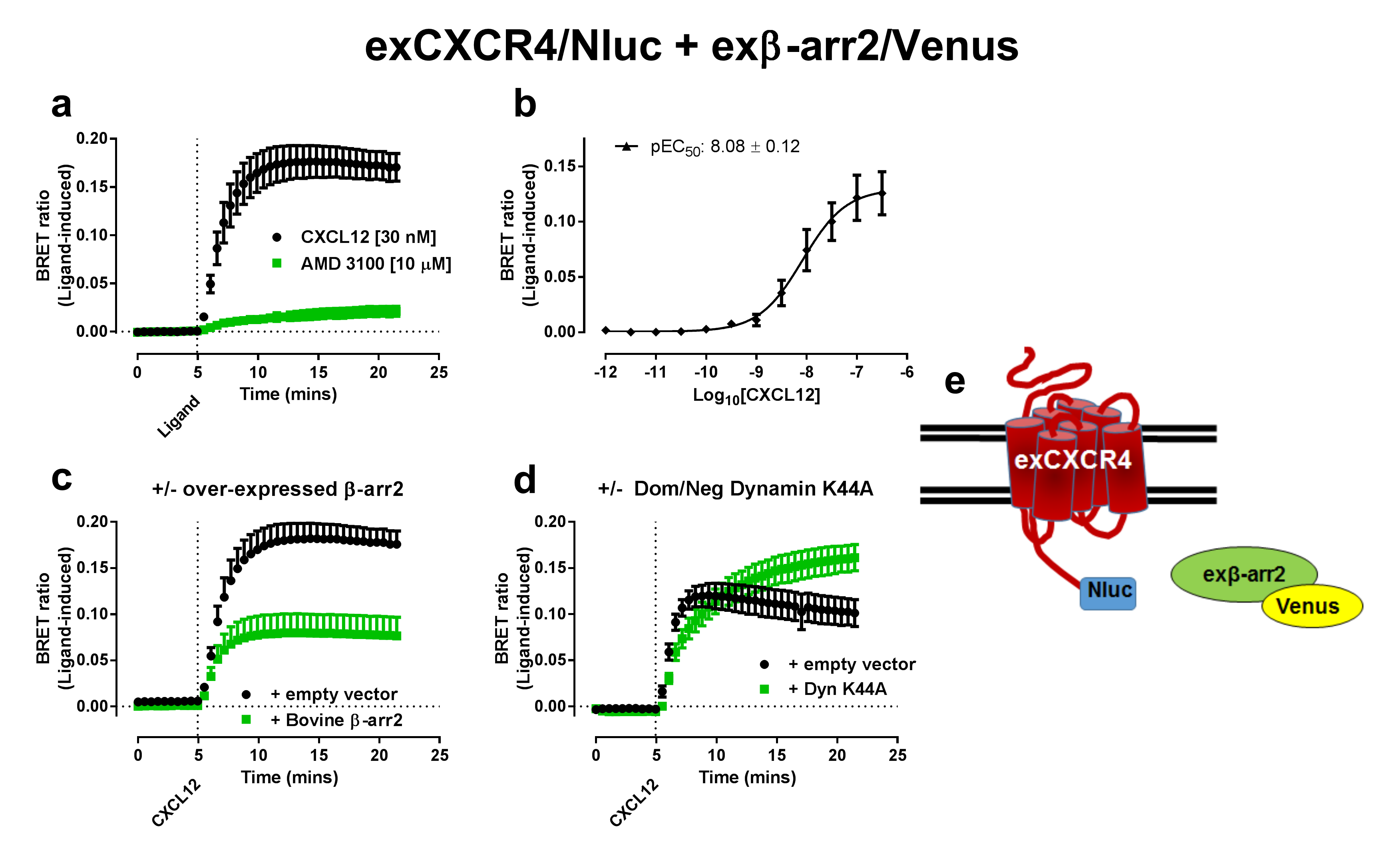
**

**Supplementary Figure 2: Monitoring β-arrestin2 recruitment to transiently-expressed CXCR4/Nluc using BRET.** HEK293FT cells transiently-transfected with cDNA coding for CXCR4/Nluc (exCXCR4/Nluc) and β-arrestin2/Venus (exβ-arr2/Venus) were used to **(a)** determine ligand-dependent CXCL12 (30 nM) recruitment of β-arrestin2 to exogenously-expressed CXCR4 in the absence or presence of the CXCR4 antagonist AMD3100 (10 µM). **(b)** Application of CXCL12 (1 pM – 300 nM) resulted in a concentration-dependent recruitment of exβ-arr2/Venus to exCXCR4/Nluc. Modulation of CXCL12 (30 nM) mediated exβ-arr2/Venus recruitment to exCXCR4/Nluc by **(c)** additional co-expression of untagged bovine β-arr2 or **(d)** dominant-negative dynamin K44A. **(e)** Schematic representation of the BRET configuration. ‘BRET ratio (ligand-induced)’ was calculated as described in *Methods.* Points represent mean ± S.E.M. of three (**d**), four (**b and c**) or five (**a**) independent experiments. (**b**) Concentration-response curve fit by non-linear regression, with points representing BRET response at approximately 2 mins (corresponding to the maximum response observed for genome-edited CXCR4/Nluc) following exposure to CXCL12 observed in a kinetic assay.

**
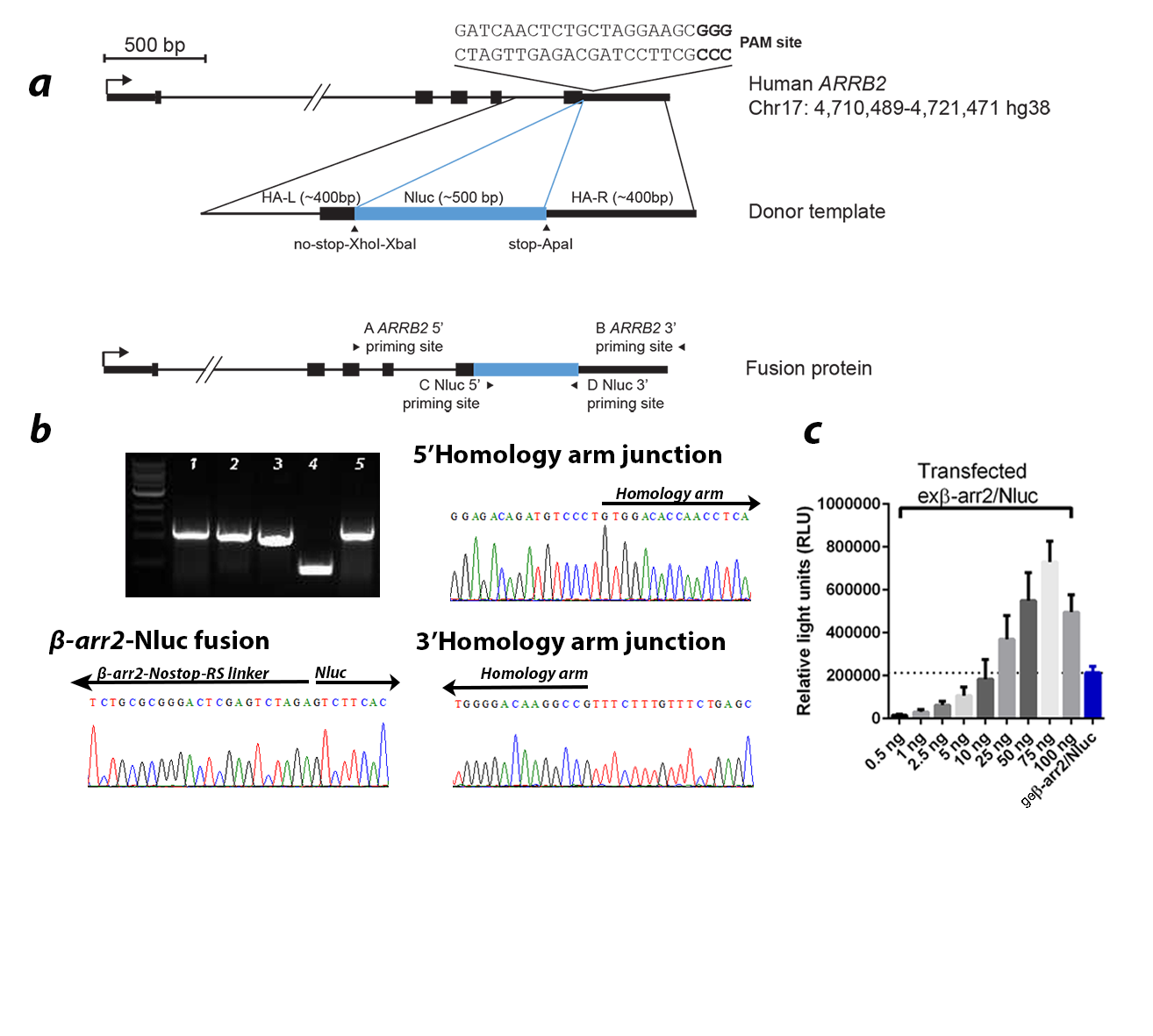
**

**Supplementary Figure 3: Generation of HEK293FT cells expressing genome-edited β-arr2/Nluc. (a)** Schematic representation of targeting the genome to fuse Nluc DNA to the end of *ARRB2* using CRISPR/Cas9-mediated homology-directed DNA repair. HA-R and HA-L are the right and left arms of the homology repair template respectively. A *ARRB2* 5′ and B *ARRB2 3*′: *ARRB2* forward and reverse priming sites. C Nluc 5′ and D Nluc 3′: Nluc forward and reverse priming sites. Rluc8 targeting was the same except with Rluc8 DNA as the insert. (**b**) Gel electrophoresis and sequencing of PCR products generated using the forward and reverse primers; A-B, lanes 1 and 5, A-D lane 2, C-B lane 3 and C-D lane 4 by amplification of genomic DNA extracted from HEK293FT cells engineered to express genome-edited β-arrestin2/Nluc lanes 1-4 or wildtype HEK293FT cells lane 5, indicating the presence of hemizygous homologous recombination at the site of interest. Sequencing was performed using bands 2 and 3. (**c**) Comparison of luminescence generated by HEK293FT cells transiently transfected with titrated amounts of cDNA coding for β-arrestin2/Nluc with luminescence generated by HEK293FT cells expressing genome-edited β-arrestin2 fused to Nluc. Bars represent mean RLU ± S.E.M. of three independent experiments.

**Supplementary Figure 4**

**
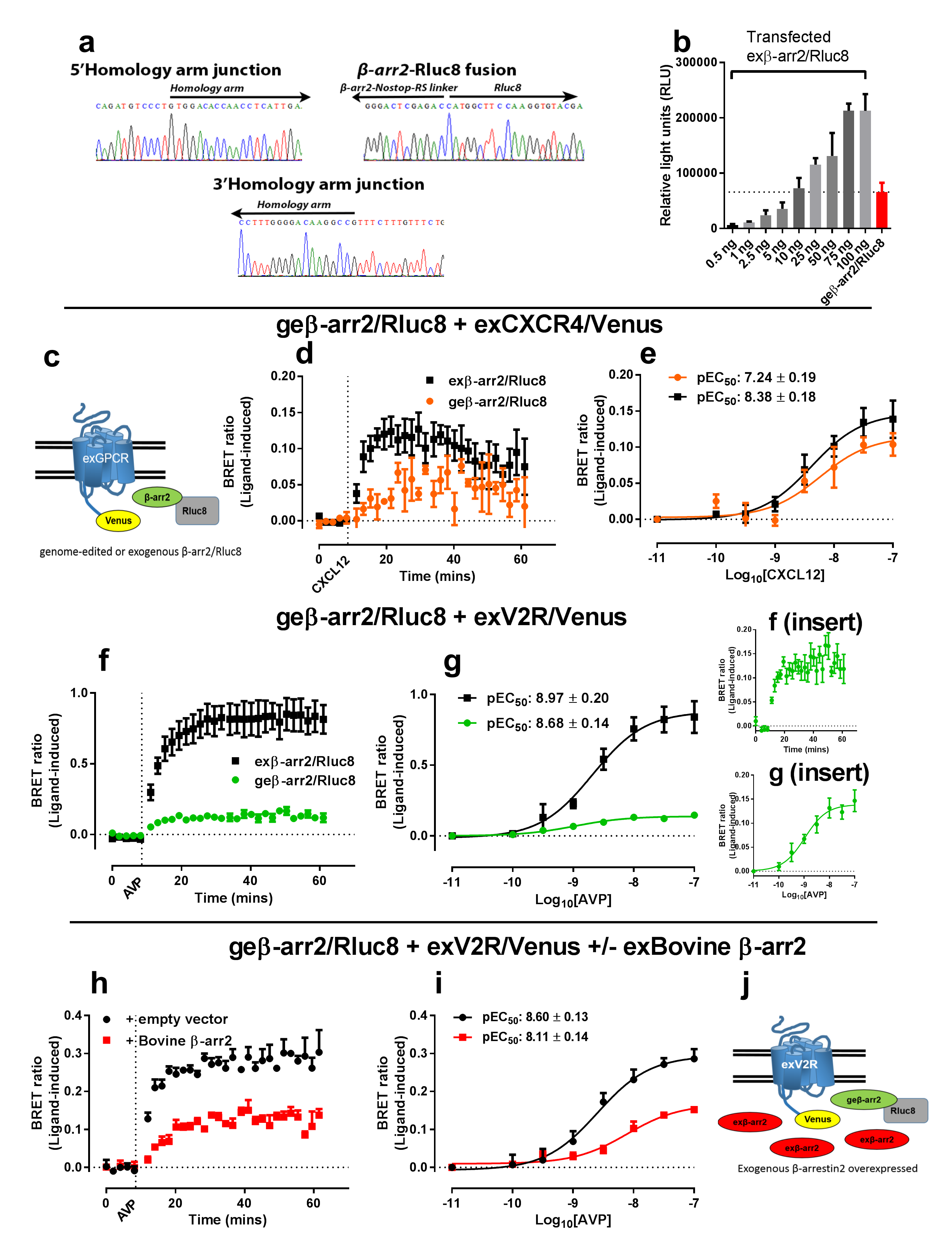
**

**Supplementary Figure 4: Investigating recruitment of genome-edited β-arrestin2 to exogenous GPCRs using Rluc8 and BRET.** (**a**) Sequencing of genomic DNA extracted from HEK293FT cells engineered to express genome-edited β-arrestin2/Rluc8. (**b**) Comparison of luminescence generated by HEK293FT cells transiently transfected with titrated amounts of cDNA coding for β-arrestin2/Rluc8 with luminescence generated by HEK293FT cells engineered to express genome-edited β-arrestin2 fused to Rluc8. (**c)** Schematic representation of the exogenously expressed GPCR fused to Venus (exGPCR/Venus) and β-arr2/Rluc8 BRET configuration. (**d-i**) HEK293FT cells expressing genome-edited β-arrestin2 fused to Rluc8 (geβ-arr2/Rluc8) transiently transfected with cDNA coding for (**d** and **e**) CXCR4 fused to Venus (exCXCR4/Venus; red circles) or (**f** and **g**) V2R fused to Venus (exV2R/Venus, green circles) as well as HEK293FT cells transiently co-transfected to express exogenous β-arrestin2 fused to Rluc8 (exβ-arr2/Rluc8, black squares) at near endogenous levels and (**d** and **e**) exCXCR4/Venus or (**f** and **g**) exV2R/Venus. (**d** and **f**) Kinetic profiles of β-arrestin2/Rluc8 recruitment initiated by addition of CXCL12 (30 nM) or AVP (100 nM) for CXCR4 and V2R respectively. Concentration-dependent recruitment of genome-edited or exogenous β-arrestin2/Rluc8 to (**e**) exCXCR4/Venus or (**g**) exV2R/Venus mediated by CXCL12 (10 pM – 100 nM) or AVP (10 pM – 100 nM) respectively. Inserts show geβ-arr2/Rluc8 recruitment to exV2R/Venus presented in **f** and **g** on expanded scale. (**h** and **i)** effect of overexpression of unlabelled exogenous β-arrestin2 (red squares) on the β-arrestin recruitment kinetic (**h**) and potency (**i**) in HEK293FT cells expressing geβ-arr2/Rluc8 transiently transfected with cDNA encoding exV2R/Venus (black circles). (**j**) Schematic representation of the BRET configuration used in **h** and **i**. Bars or points represent mean ± S.E.M. of three independent experiments, with curve fit by non-linear regression of maximum response observed over kinetic time-course. ‘BRET ratio (ligand-induced)’ was calculated as described in *Methods*.

**
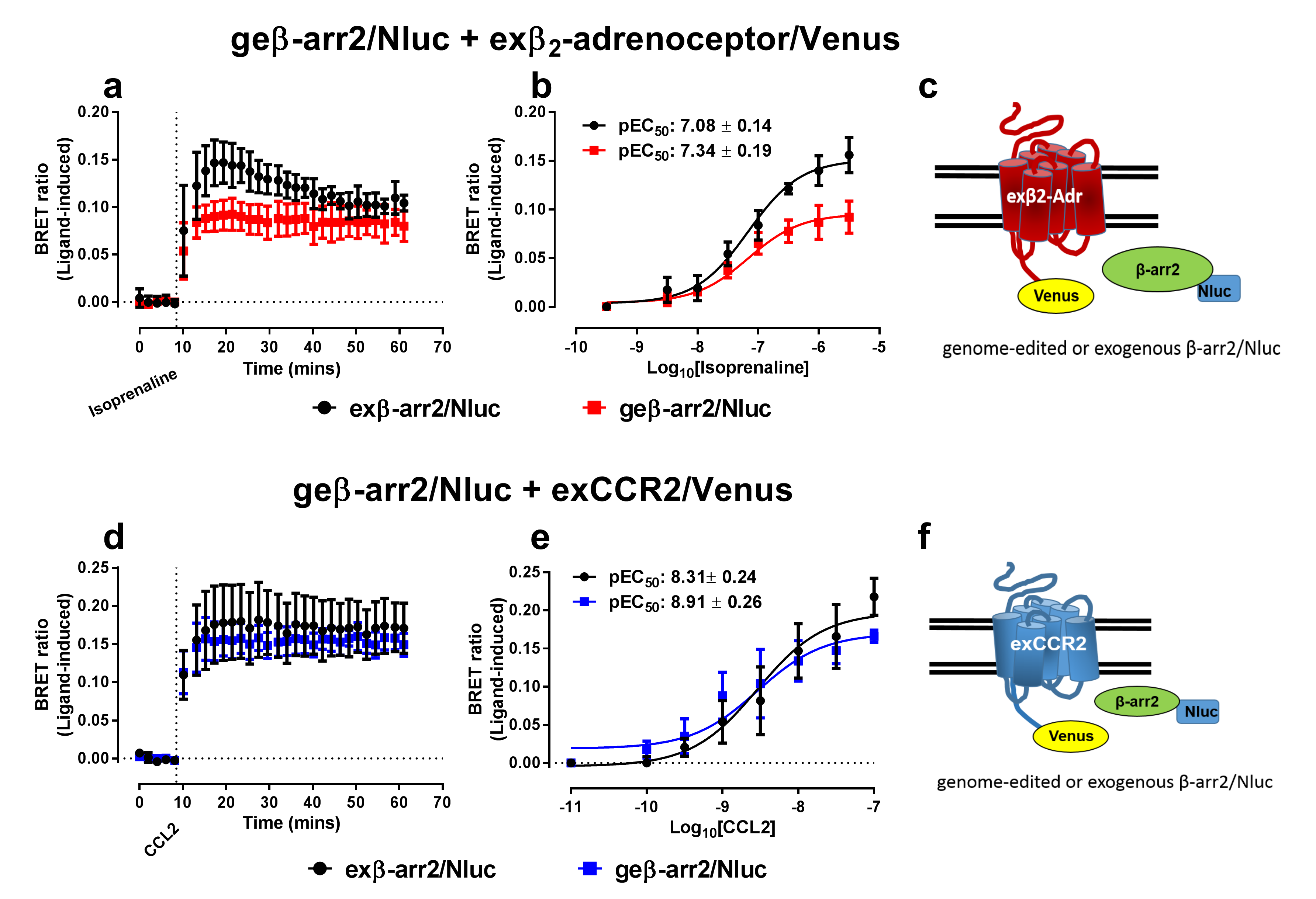
**

**Supplementary Figure 5: Investigating recruitment of genome-edited β-arrestin2 to exogenous GPCRs using Nluc and BRET.** HEK293FT cells expressing genome-edited β-arrestin2 fused to Nluc (geβ-arr2/Nluc) transiently transfected with cDNA coding for (**a-c**) the β_2_-adrenoceptor fused to Venus (exβ_2_-adrenoceptor/Venus; red squares) or (**d-f**) CCR2 fused to Venus (exCCR2/Venus, blue squares) as well as HEK293FT cells transiently co-transfected to express exogenous β-arrestin2 fused to Nluc (exβ-arr2/Nluc, black circles) at near endogenous levels and (**a-c**) exβ_2_-adrenoceptor/Venus or (**d-f**) exCCR2/Venus. (**a** and **d**) Kinetic profiles of β-arrestin2/Nluc recruitment initiated by addition of isoprenaline (10 µM) or CCL2 (100 nM) for β_2_-adrenoceptors and CCR2 respectively. Concentration-dependent recruitment of genome-edited or exogenous β-arrestin2/Nluc to (**b**) exβ_2_-adrenoceptor/Venus or (**e**) exCCR2/Venus mediated by isoprenaline (30 pM – 3 µM) or CCL2 (10 pM – 100 nM) respectively. (**c** and **f)** Schematic representations of the BRET configurations used. ‘BRET ratio (ligand-induced)’ was calculated as described in *Methods*. Points represent mean ± S.E.M. of maximum response observed in a kinetic time-course in three independent experiments, with curve fit by non-linear regression.

**
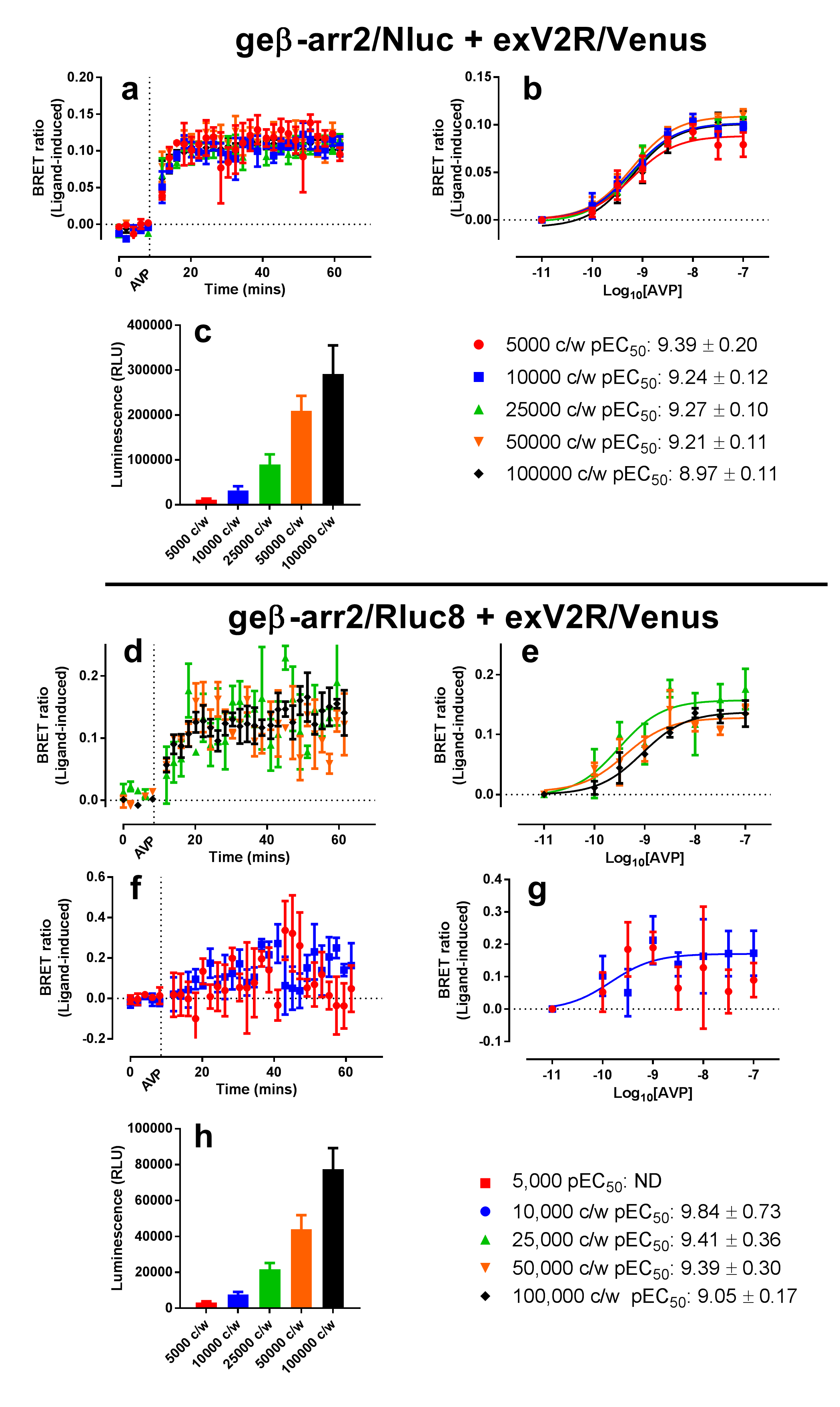
**

**Supplementary Figure 6: Determining luciferase sensitivity by cell number titration.** HEK293FT cells expressing genome-edited β-arrestin2 fused to Nluc (**a-c**; geβ-arr2/Nluc) or Rluc8 (**d-h**; geβ-arr2/Rluc8) were transiently transfected with cDNA coding for V2R fused to Venus (exV2R/Venus) and used to determine the effect of cell number on the (**a, d** and **f**) kinetic of genome-edited β-arrestin2/luciferase recruitment to exV2R/Venus mediated by AVP (30 nM) as well as (**b, e** and **g**) potency of AVP (10 pM -100 nM) mediated responses. (**c** and **h**) luminescence as relative light units (RLU) detected for each cell number titration. c/w, cells/well; ND, not determined. Points or bars represent mean ± S.E.M. of three independent experiments, with curve fit by non-linear regression of maximum response observed over kinetic time-course. ‘BRET ratio (ligand-induced)’ was calculated as described in *Methods.* Luminescence value is the last baseline read before the addition of AVP measured through the 460–490 nm filter.

**
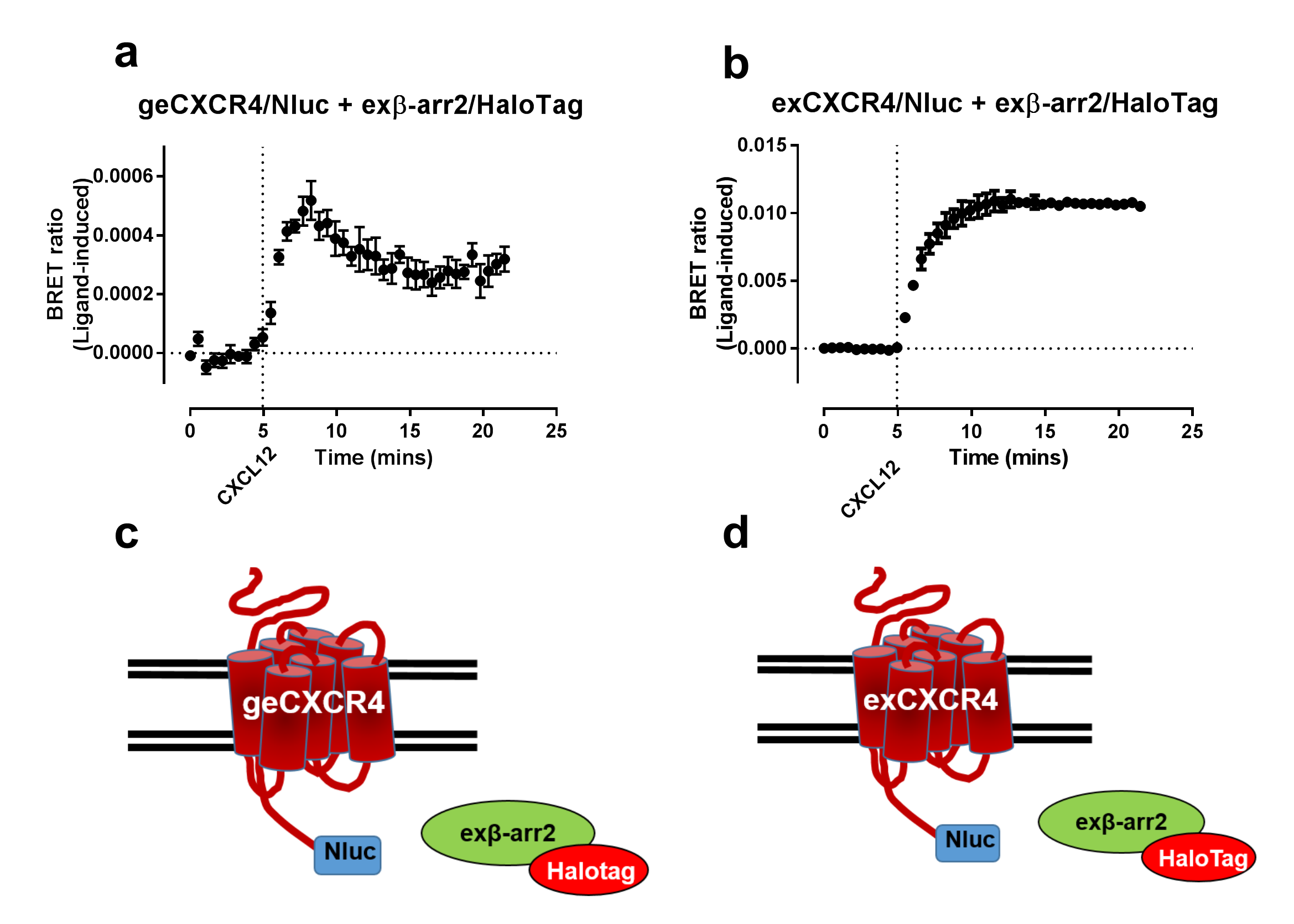
**

**Supplementary Figure 7: Comparison of genome-edited CXCR4/Nluc versus transiently expressed CXCR4/Nluc recruiting β-arrestin2/HaloTag monitored using NanoBRET. (a)** HEK293FT cells expressing genome-edited CXCR4 fused to Nluc (geCXCR4/Nluc) transiently transfected with cDNA coding for β-arrestin2/HaloTag (exβ-arr2/HaloTag) and or **(b)** HEK293FT cells transiently transfected with cDNA coding for CXCR4 fused to Nluc (exCXCR4/Nluc) and β-arrestin2/HaloTag (exβ-arr2/HaloTag) were used to determine the effect of changing the BRET acceptor on the kinetic profile when monitoring CXCL12 (100 nM) induced recruitment of β-arrestin2 to CXCR4. The NCT ligand was used in combination with the HaloTag (see *Methods*). **(c** and **d)** Schematic representations of the BRET configuration corresponding to **a** and **b** respectively. ‘BRET ratio (ligand-induced)’ was calculated as described in *Methods.* Points represent mean ± S.E.M. of five (**a**) or two (**b**) independent experiments.

**
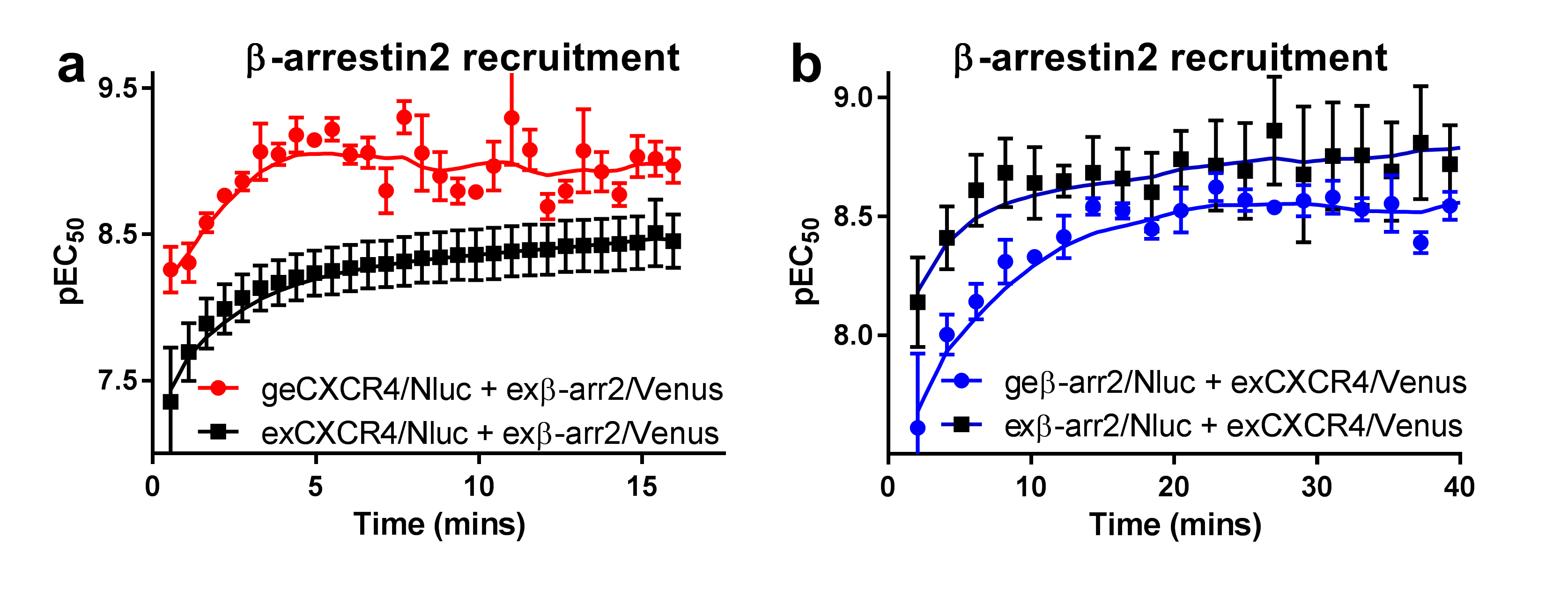
**

**Supplementary Figure 8: Effect of BRET configuration and/or kinetics on the quantification of potency.** Potency (pEC_50_) of CXCL12-mediated β-arrestin2 recruitment to CXCR4 was quantified over the kinetic time course using: (**a**) HEK293FT cells expressing genome-edited CXCR4 fused to Nluc (geCXCR4/Nluc) transfected with exogenous β-arrestin2/Venus (exβ-arr2/Venus) (red circles), or HEK293FT cells transiently transfected to express both exCXCR4/Nluc and exβ-arr2/Venus (black squares); or (**b**) HEK293FT cells expressing genome-edited β-arrestin2 fused to Nluc (geβ-arr2/Nluc) transfected with exogenous CXCR4/Venus (exCXCR4/Venus) (blue circles) or HEK293FT cells transiently transfected to express both exCXCR4/Venus and exβ-arr2/Nluc (black squares). Points represent mean ± S.E.M. of three (**a,** black squares only; **b**) or four (**a**, red circles only) independent experiments. pEC_50_ values calculated using GraphPad Prism from non-linear regression analysis of the BRET (ligand-induced) response observed in a kinetic time-course following ligand addition at time zero. Trend lines fitted with GraphPad Prism using Lowess curve fitting. ‘BRET ratio (ligand-induced)’ was calculated as described in *Methods.*


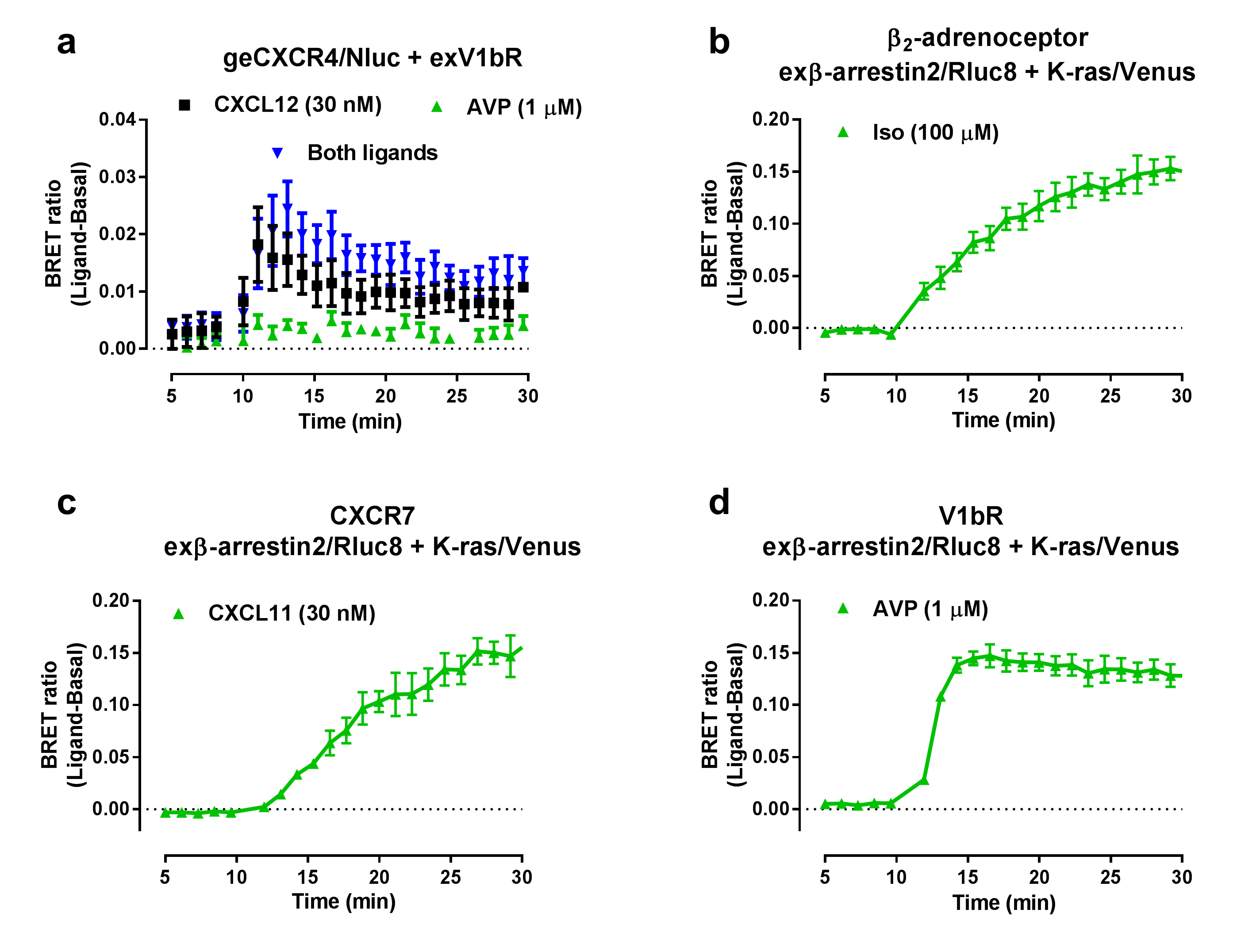


**Supplementary Figure 9: GPCR-HIT control experiments.** (**a**) HEK293FT cells expressing genome-edited CXCR4 fused to Nluc (geCXCR4/Nluc) transiently transfected with cDNA coding for β-arrestin2/Venus and Vasopressin V1b receptor were used to carry out BRET assays using the GPCR-HIT configuration. (**b-d**) Wildtype HEK293FT cells transfected with β-arrestin2/Rluc8 and K-ras/Venus, as well as (**b**) β_2_-adrenoceptor (**c**) CXCR7 or (**d**) Vasopressin V1b receptor to monitor bystander BRET. In (**a**), cells were stimulated with CXCL12 (30 nM, black squares), AVP (100 µM, green upward triangles) or both ligands simultaneously (blue downward triangles). In (**b-d**), cells were stimulated with isoprenaline (100 µM), CXCL11 (30 nM) or AVP (1 µM) respectively. ‘BRET ratio (ligand-induced)’ was calculated as described in *Methods.* Points represent mean BRET ratio ± S.E.M. Ligand addition at time 10 minutes. Data generated from four independent experiments.


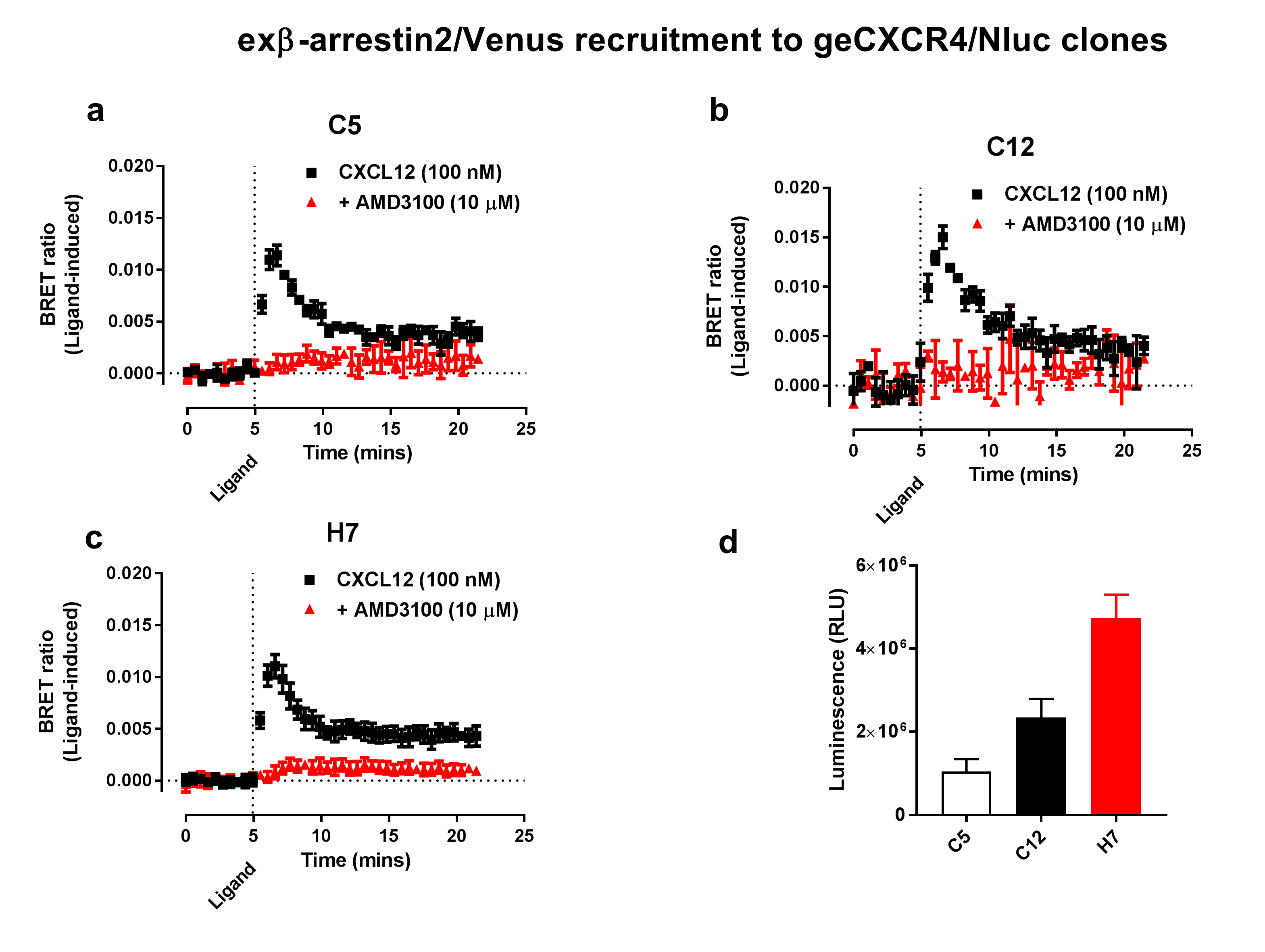


**Supplementary Figure 10: Monitoring β-arrestin2 recruitment to genome-edited CXCR4/Nluc using BRET.** HEK293FT clones C5 (**a**), C12 (**b**) and H7 (**c**) expressing genome-edited CXCR4 fused to Nluc (geCXCR4/Nluc) transiently transfected with cDNA coding for β-arrestin2/Venus (exβ-arrestin2/Venus) were used to determine CXCL12 (100 nM)-induced recruitment of β-arrestin2 to CXCR4 in the absence or presence of the CXCR4 antagonist AMD3100 (10 µM). **(d)** Comparison of the luminescence generated by each clone in parallel on the CLARIOstar. ‘BRET ratio (ligand-induced)’ was calculated as described in *Methods.* Points represent mean ± S.E.M. of two (red points in b), three (a and black points in b) or four (c) independent experiments. Note panel c is a direct replication of Figure 1a and is included here for comparison purposes only.


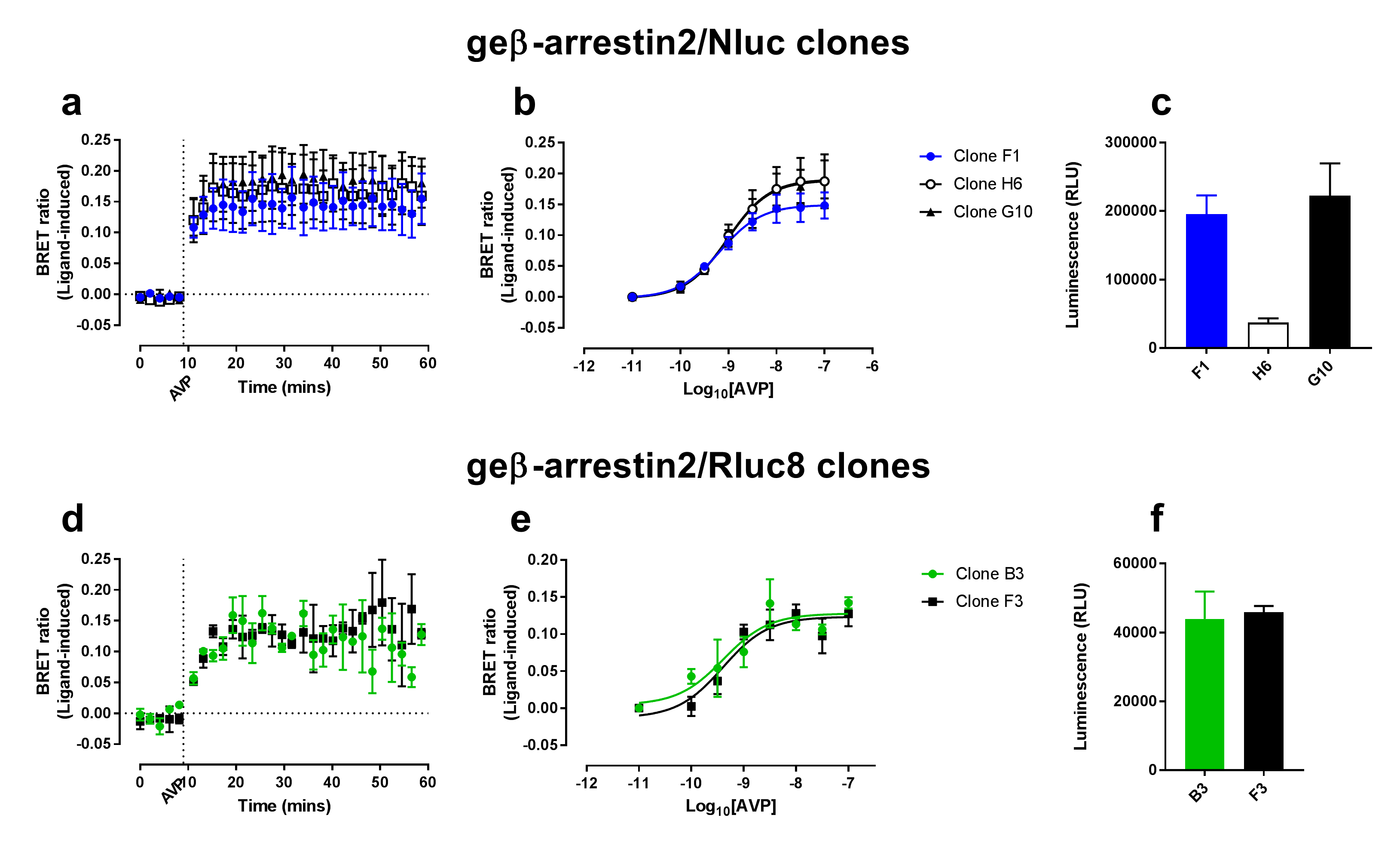


**Supplementary Figure 11: Comparison of genome-edited β-arrestin2/luciferase clones in BRET recruitment assays.** HEK293FT clones F1, H6 and G10 expressing genome-edited β-arrestin2 fused to Nluc (geβ-arrestin2/Nluc) (**a-c**) or HEK293FT clones B3 and F3 expressing genome-edited β-arr2 fused to Rluc8 (geβ-arrestin2/Rluc8) transiently transfected with cDNA coding for V2R/Venus (exV2R/Venus) were used to **(a,d)** determine ligand-dependent AVP (100 nM) recruitment of β-arrestin2 to exV2R/Venus. **(b,e)** Concentration-dependent recruitment of genome-edited or exogenous β-arrestin2/luciferase to exV2R/Venus mediated by AVP (10 pM – 100 nM). (**c,f**) Comparison of the luminescence generated by each clone in parallel. ‘BRET ratio (ligand-induced)’ was calculated as described in *Methods.* Points represent mean ± S.E.M. of three or four independent experiments using a seeding density of 50,000 cells/well. Note blue points and bars in a-c (clone F1) and green points and bars in d-f (clone B3) are direct replications from Figure 2d and e and from supplementary figures 6d-e and 6h, and are included here for comparison purposes only.

**Supplementary table** 1: sgRNA sequences

| sgRNA target | sgRNA |
| --- | --- |
| *ARRB2* | GATCAACTCTGCTAGGAAGC**GGG** |
| *CXCR4* | *g*ACTTGAAGACTCAGACTCAG**TGG** |

sgRNA sequences upstream of protospacer adjacent motif **PAM (bold)** cloned into the px459 plasmid, *g*; additional guanine added for efficient transcription from the U6 promoter

**Supplementary table 2: *CXCR4* donor plasmid repair sequence**

| ***CXCR4*/Nluc repair template sequence** |
| --- |
| [TCTTTGTCATCACGCTTCCCTTCTGGGCAGTTGATGCCGTGGCAAACTGGTACTTTGGGAACTTCCTATGCAAGGCAGTCCATGTCATCTACACAGTCAACCTCTACAGCAGTGTCCTCATCCTGGCCTTCATCAGTCTGGACCGCTACCTGGCCATCGTCCACGCCACCAACAGTCAGAGGCCAAGGAAGCTGTTGGCTGAAAAGGTGGTCTATGTTGGCGTCTGGATCCCTGCCCTCCTGCTGACTATTCCCGACTTCATCTTTGCCAACGTCAGTGAGGCAGATGACAGATATATCTGTGACCGCTTCTACCCCAATGACTTGTGGGTGGTTGTGTTCCAGTTTCAGCACATCATGGTTGGCCTTATCCTGCCTGGTATTGTCATCCTGTCCTGCTATTGCATTATCATCTCCAAGCTGTCACACTCCAAGGGCCACCAGAAGCGCAAGGCCCTCAAGACCACAGTCATCCTCATCCTGGCTTTCTTCGCCTGTTGGCTGCCTTACTACATTGGGATCAGCATCGACTCCTTCATCCTCCTGGAAATCATCAAGCAAGGGTGTGAGTTTGAGAACACTGTGCACAAGTGGATTTCCATCACCGAGGCCCTAGCTTTCTTCCACTGTTGTCTGAACCCCATCCTCTATGCTTTCCTTGGAGCCAAATTTAAAACCTCTGCCCAGCACGCACTCACCTCTGTGAGCAGAGGGTCCAGCCTCAAGATCCTCTCCAAAGGAAAGCGAGGTGGACATTCATCTGTTTC***g***A**CTGAGTCTGAGTCTTCAAGT**TTTCACTCCAGC]˂**GCG**GGA*CTCGAG***GTCTTCACACTCGAAGATTTCGTTGGGGACTGGCGACAGACAGCCGGCTACAACCTGGACCAAGTCCTTGAACAGGGAGGTGTGTCCAGTTTGTTTCAGAATCTCGGGGTGTCCGTAACTCCGATCCAAAGGATTGTCCTGAGCGGTGAAAATGGGCTGAAGATCGACATCCATGTCATCATCCCGTATGAAGGTCTGAGCGGCGACCAAATGGGCCAGATCGAAAAAATTTTTAAGGTGGTGTACCCTGTGGATGATCATCACTTTAAGGTGATCCTGCACTATGGCACACTGGTAATCGACGGGGTTACGCCGAACATGATCGACTATTTCGGACGGCCGTATGAAGGCATCGCCGTGTTCGACGGCAAAAAGATCACTGTAACAGGGACCCTGTGGAACGGCAACAAAATTATCGACGAGCGCCTGATCAACCCCGACGGCTCCCTGCTGTTCCGAGTAACCATCAACGGAGTGACCGGCTGGCGGCTGTGCGAACGCATTCTGGCGTAA***TCTAGAGGGCCC>*[CACAGATGTAAAAGACTTTTTTTTATACGATAAATAACTTTTTTTTAAGTTACACATTTTTCAGATATAAAAGACTGACCAATATTGTACAGTTTTTATTGCTTGTTGGATTTTTGTCTTGTGTTTCTTTAGTTTTTGTGAAGTTTAATTGACTTATTTATATAAATTTTTTTTGTTTCATATTGATGTGTGTCTAGGCAGGACCTGTGGCCAAGTTCTTAGTTGCTGTATGTCTCGTGGTAGGACTGTAGAAAAGGGAACTGAACATTCCAGAGCGTGTAGTGAATCACGTAAAGCTAGAAATGATCCCCAGCTGTTTATGCATAGATAATCTCTCCATTCCCGTGGAACGTTTTTCCTGTTCTTAAGACGTGATTTTGCTGTAGAAGATGGCACTTATAACCAAAGCCCAAAGTGGTATAGAAATGCTGGTTTTTCAGTTTTCAGGAGTGGGTTGATTTCAGCACCTACAGTGTACAGTCTTGTATTAAGTTGTTAATAAAAGTACATGTTAAACTTACTTAGTGTTATGTTCTGATTTCTGTTGACATTCTTTTGCTAGTAGAAGACAAAAGTAATACATTTATGGTATGCAAAGCACTATCCTAGGTATTTCATTGTAATATTTTACTTACCCCTTATCACAACTCTGATAGATTCTGCTTCTGTTACTAATTACATTTTATAGAAGAGGAAACGGAGGCACAGAAAGCCTAAGTAACTTGGTTAAAGGCATGTAGTAAGTATCAAATCCTGTATTTTAAACCAGGTAACATGACTTAACGAATCTGAAGCCTTC]  [SEQUENCE]; 800bp left and right homology arms, ***g***; introduction silent mutation of PAM site repair template, **CTGAGTCTGAGTCTTCAAGT;** sgRNA binding site, <SEQUENCE>: sequence inserted, **GCG; CXCR4 stop-Ala mutation**, GGA*CTCGAG*; linker-*XhoI restriction site*, **SEQUENCE; Nluc insert**, *TCTAGAGGGCCC*; *XbaI-ApaI restriction site* |

Donor plasmid was synthesised by GeneArt (Invitrogen).

**Supplementary table 3: *ARRB2* donor plasmid repair sequences**

| ***β-arrestin2* repair template sequence** |
| --- |
| [GTGGACACCAACCTCATTGAATTTGATACCAAGTAAGAAACTCATTCCCCTACTTGACCCTCTTGGGACAAAGATTCCTATAACATTCAAATCTGCCCTCATACCTCTTCCTTGCTTTTGGTGGGGAGAAGCGGATTGTAGCATCAAATCAAGATGCCTTAGCCTTGTGAGGCTGCCTCTTGCTGCCTTTTCTTTGTCCCTTCCTGTAAATACCTCTGGTCCCACTGCTGTTCGAACGCCTCTGTCCCAGAGGCCTAGCTTCGGGGAGGGCAGGGAGTGGGAGGCTGGGACAAGAGTCAGAAGCCCTCACCTCACAACCCTCTTTCCCACCACCAAGCTATGCCACAGATGATGACATTGTGTTTGAGGACTTTGCCCGGCTTCGGCTGAAGGGGATGAAGGATGACGACTATGATGATCAACTCTGC]<**GCG**GGA*CTCGAG*~~~LUCIFERASE_STOP~~~~*TCTAGAGGGCCC*>[GAAGC**GGG**GTGGGAAGAAGGGAGGGGATGGGGTTGGGAGAGGTGAGGGCAGGATTAAGATCCCCACTGTCAATGGGGGATTGTCCCAGCCCCTCTTCCCTTCCCCTCACCTGGAAGCTTCTTCAACCAATCCCTTCACACTCTCTCCCCCATCCCCCCAAGATACACACTGGACCCTCTCTTGCTGAATGTGGGCATTAATTTTTTGACTGCAGCTCTGCTTCTCCAGCCCCGCCGTGGGTGGCAAGCTGTGTTCATACCTAAATTTTCTGGAAGGGGACAGTGAAAAGAGGAGTGACAGGAGGGAAAGGGGGAGACAAAACTCCTACTCTCAACCTCACACCAACACCTCCCATTATCACTCTCTCTGCCCCCATTCCTTCAAGAGGAGACCCTTTGGGGACAAGGCCG]  SEQUENCE; left and right homology arms 400bp, <SEQUENCE>: sequence inserted, **GCG; *ARRB2* stop-Ala mutation**, GGA*CTCGAG*; linker-*XhoI restriction site*, **~~~LUCIFERASE_STOP~~~**, insert site of luciferase-stop codon, *TCTAGAGGGCCC*; *–XbaI-ApaI restriction site,* ***GGG; site of PAM*** |

N.B.: Donor plasmid [homology arms] were synthesised by GeneArt (Invitrogen). Nluc and Rluc8 were sub-cloned into the donor plasmid containing the homology arms using the XhoI, XbaI and ApaI restriction site cluster and conventional cloning methods.

**Supplementary table 4: Sequences of Rluc8 and Nluc inserts cloned into the *ARRB2* donor plasmid**

| ***Rluc8* sequence** |
| --- |
| GGA*CTCGAGACC*ATGGCTTCCAAGGTGTACGACCCCGAGCAACGCAAACGCATGATCACTGGGCCTCAGTGGTGGGCTCGCTGCAAGCAAATGAACGTGCTGGACTCCTTCATCAACTACTATGATTCCGAGAAGCACGCCGAGAACGCCGTGATTTTTCTGCATGGTAACGCTACCTCCAGCTACCTGTGGAGGCACGTCGTGCCTCACATCGAGCCCGTGGCTAGATGCATCATCCCTGATCTGATCGGAATGGGTAAGTCCGGCAAGAGCGGGAATGGCTCATATCGCCTCCTGGATCACTACAAGTACCTCACCGCTTGGTTCGAGCTGCTGAACCTTCCAAAGAAAATCATCTTTGTGGGCCACGACTGGGGGGCTGCTCTGGCCTTTCACTACGCCTACGAGCACCAAGACAGGATCAAGGCCATCGTCCATATGGAGAGTGTCGTGGACGTGATCGAGTCCTGGGACGAGTGGCCTGACATCGAGGAGGATATCGCCCTGATCAAGAGCGAAGAGGGCGAGAAAATGGTGCTTGAGAATAACTTCTTCGTCGAGACCGTGCTCCCAAGCAAGATCATGCGGAAACTGGAGCCTGAGGAGTTCGCTGCCTACCTGGAGCCATTCAAGGAGAAGGGCGAGGTTAGACGGCCTACCCTCTCCTGGCCTCGCGAGATCCCTCTCGTTAAGGGAGGCAAGCCCGACGTCGTCCAGATTGTCCGCAACTACAACGCCTACCTTCGGGCCAGCGACGATCTGCCTAAGCTGTTCATCGAGTCCGACCCTGGGTTCTTTTCCAACGCTATTGTCGAGGGAGCTAAGAAGTTCCCTAACACCGAGTTCGTGAAGGTGAAGGGCCTCCACTTCCTCCAGGAGGACGCTCCAGATGAAATGGGTAAGTACATCAAGAGCTTCGTGGAGCGCGTGCTGAAGAACGAGCAGTAA*TCTAGAGGGCCC*  GCGGGA*CTCGAGACC*; linker-*XhoI restriction site-linker*, **SEQUENCE; Rluc8 insert-Stop**, *TCTAGAGGGCCC,* *XbaI-ApaI restriction site* |
| ***Nluc* sequence** |
| GGA*CTCGAG***GTCTTCACACTCGAAGATTTCGTTGGGGACTGGCGACAGACAGCCGGCTACAACCTGGACCAAGTCCTTGAACAGGGAGGTGTGTCCAGTTTGTTTCAGAATCTCGGGGTGTCCGTAACTCCGATCCAAAGGATTGTCCTGAGCGGTGAAAATGGGCTGAAGATCGACATCCATGTCATCATCCCGTATGAAGGTCTGAGCGGCGACCAAATGGGCCAGATCGAAAAAATTTTTAAGGTGGTGTACCCTGTGGATGATCATCACTTTAAGGTGATCCTGCACTATGGCACACTGGTAATCGACGGGGTTACGCCGAACATGATCGACTATTTCGGACGGCCGTATGAAGGCATCGCCGTGTTCGACGGCAAAAAGATCACTGTAACAGGGACCCTGTGGAACGGCAACAAAATTATCGACGAGCGCCTGATCAACCCCGACGGCTCCCTGCTGTTCCGAGTAACCATCAACGGAGTGACCGGCTGGCGGCTGTGCGAACGCATTCTGGCGTAA***TCTAGAGGGCCC*  GGA*CTCGAG*; linker-*XhoI restriction site*, **SEQUENCE; No-start-Nluc insert-Stop**, *TCTAGAGGGCCC*; *XbaI-ApaI restriction site* |

Luciferase fragments were sub-cloned into the *ABRR2* donor plasmid using the XhoI, XbaI, ApaI restriction site cluster.

**Supplementary table 5: G**enomic DNA sequencing primers

|  | *Forward primer* | *Reverse primer* |
| --- | --- | --- |
| *CXCR4* | CTGAGAAGCATGACGGACAA | ATTGCACAGACCTGGGTTTC |
| *ARRB2* | AGACCCCAGTCAGGTGAG | GGGTGCAGAAGAGAGACTGG |
| *Nluc* | GGAGGTGTGTCCAGTTTGTT | GTCACTCCGTTGATGGTTACTC |
| *Rluc8* | GCTGGACTCCTTCATCAACTAC | CTCCCTTAACGAGAGGGATCT |

***N.B.:*** *CXCR4* and *ARRB2* bind outside the homology arms of the donor template

**Supplementary table 6**: Optimised plate reader parameters

| **Figure** | **Plate reader** | **Emission (nm) and gain parameters** |
| --- | --- | --- |
| Figure 1 | CLARIOstar | Em: 570-100; Gain 3400  Em: 450-60; Gain 2800 |
| Figure 2 | LUMIstar | Em: 535-15; Gain 3200  Em: 475-30; Gain 2600 |
| Figure 3a and b | LUMIstar | Em: 535-15; Gain 3200  Em: 475-30; Gain 2600 |
| Figure 3c and d | LUMIstar | Em: 535-15; Gain 3600  Em: 475-30; Gain 3200 |
| Figure4 a-f | CLARIOstar | Em: 570-100; Gain 3400  Em: 450-60; Gain 2000 |
| Figure 4h | CLARIOstar | Em: 660-100; Gain 3600  Em: 450-60; Gain 3200  Em: 550-60; Gain 3600 |
| Figure 5 | LUMIstar | Em: 535-15; Gain 3600  Em: 475-30; Gain 3200 |
| Supplementary Figure 1c | LUMIstar | Em: 535-15; Gain 3600  Em: 475-30; Gain 3000 |
| Supplementary Figure 1d | LUMIstar | Em: 535-15; Gain 3200  Em: 475-30; Gain 2600 |
| Supplementary  Figure 1e | CLARIOstar | Em: 450-60; Gain 3600 |
| Supplementary  Figure 1f | CLARIOstar  Fluorescence Intensity | Ex: 345-365 nm  Em: 440-460 |
| Supplementary Figure 2 | CLARIOstar | Em: 570-100; Gain 3000  Em: 450-60; Gain 2400 |
| Supplementary Figure 3c | LUMIstar | Em: 535-15; Gain 3200  Em: 475-30; Gain 2600 |
| Supplementary Figure 4 | LUMIstar | Em: 535-15; Gain 3600  Em: 475-30; Gain 3200 |
| Supplementary Figure 5 | LUMIstar | Em: 535-15; Gain 3200  Em: 475-30; Gain 2600 |
| Supplementary Figure 6a-c | LUMIstar | Em: 535-15; Gain 3200  Em: 475-30; Gain 2600 |
| Supplementary Figure 6d-h | LUMIstar | Em: 535-15; Gain 3600  Em: 475-30; Gain 3200 |
| Supplementary  Figure 7a | CLARIOstar | Em: >610; Gain 3600  Em: 450-60; Gain 3200 |
| Supplementary  Figure 7b | CLARIOstar | Em: >610; Gain 3600  Em: 450-60; Gain 3000 |
| Supplementary  Figure 8a | CLARIOstar | Em: 570-100; Gain 3400  Em: 450-60; Gain 2800 |
| Supplementary  Figure 8b | LUMIstar | Em: 535-15; Gain 3200  Em: 475-30; Gain 2600 |
| Supplementary Figure 9 | LUMIstar | Em: 535-15; Gain 3600  Em: 475-30; Gain 3200 |
| Supplementary  Figure 10 | CLARIOstar | Em: 570-100; Gain 3400  Em: 450-60; Gain 2800 |
| Supplementary  Figure 11 | LUMIstar | Em: 535-15; Gain 3200  Em: 475-30; Gain 2600 |
| Supplementary  Figure 11 | LUMIstar | Em: 535-15; Gain 3600  Em: 475-30; Gain 3200 |
